# Nature-based solutions for nutrient and emerging contaminant reduction in a Mediterranean Ramsar wetland

**DOI:** 10.1007/s10653-026-03377-4

**Published:** 2026-08-08

**Authors:** Emilio Moreno, María Gómez-Pozuelos, Francisco Guerrero, Cristina Postigo, África Lupión, José María Conde-Porcuna, Inmaculada de Vicente

**Affiliations:** 1 Institute of Water Research, University of Granada, Ramón y Cajal, 4, 18071 Granada, Spain; 2 Department of Animal Biology, Plant Biology and Ecology, University of Jaén, Campus Las Lagunillas s/n, 23071 Jaén, Spain; 3 Center for Advanced Studies in Earth Sciences, Energy and Environment, University of Jaén, Campus Las Lagunillas s/n, 23071 Jaén, Spain; 4 Department of Civil Engineering, School of Civil Engineering, University of Granada, Avenida de Fuentenueva s/n, 18071 Granada, Spain; 5 Consejería de Sostenibilidad, Medio Ambiente y Economía Azul (Regional Ministry of Sustainability, Environment and Blue Economy), Reserva Natural Laguna Fuente de Piedra (Fuente de Piedra Nature Reserve), 29520 Fuente de Piedra, Spain; 6 Department of Ecology, University of Granada, Avenida de Fuentenueva s/n, 18071 Granada, Spain

**Keywords:** Eutrophication, Nature-based solutions, Water quality, Constructed wetlands, Emerging contaminants, Ecotoxicological risk, Water circulation

## Abstract

Constructed wetlands are widely recognized as nature-based solutions for improving water quality in systems receiving treated wastewater. However, their effectiveness depends primarily on hydrological connectivity, contaminant properties, and hydraulic residence time. In Mediterranean wetlands, where strong hydrological variability and seasonal water scarcity influence water quality dynamics, understanding attenuation processes under contrasting water-circulation scenarios is relevant for protecting sensitive downstream ecosystems. This study assessed the capacity of a semi-natural pond system i.e., a series of interconnected shallow ponds comprising both natural and managed water bodies that receive treated wastewater while retaining natural wetland characteristics, located upstream of the Fuente de Piedra Ramsar wetland main basin (southern Spain) to reduce nutrients and emerging contaminants under contrasting water-circulation scenarios. Across 26 sampling events, substantial reductions were observed when treated wastewater flowed through the semi-natural pond system before reaching the main wetland basin, including decreases of 75% in total nitrogen. Marked attenuation was detected for emerging contaminants with shorter environmental half-lives, such as trimethoprim, naproxen, caffeine and selected triazine metabolites showed removals exceeding 70–90%, whereas more persistent substances, including carbamazepine and gemfibrozil, exhibited limited reduction. In contrast, negligible changes occurred during a temporary bypass configuration put in place for restoration works, in which treated wastewater avoided most of the semi-natural ponds before reaching the main wetland basin. Dry-season conditions enhanced contaminant attenuation, largely reflecting lower flows and extended hydraulic residence times. Cumulative ecotoxicological risk, estimated using risk quotients (RQ_sample_) based on measured environmental concentrations and the lowest predicted no-effect concentrations for freshwater organisms, declined downstream under the regular flow pathway, particularly for pesticides, but moderate risk persisted under the bypass scenario. Overall, the semi-natural pond system demonstrates a strong but hydrologically dependent capacity to mitigate nutrient and emerging contaminant loads entering a Mediterranean Ramsar wetland. Nevertheless, elevated phosphorus concentrations and the persistence of certain emerging contaminants highlight the limitations of natural attenuation alone and support the integration of complementary management and treatment strategies to ensure long-term ecosystem protection.

**Supplementary Information:**

The online version contains supplementary material available at https://doi.org/10.1007/s10653-026-03377-4.

## Introduction

Despite covering only 1.5% of the Earth’s surface, wetlands are recognized as critical drivers of biodiversity conservation and rural socioeconomic development globally (Mitsch & Gossilink, 2000). Wetlands provide 40% of ecosystem services worldwide, such as provisioning, regulating, habitat, and cultural services (Millenium Ecosystem Assessment, 2005; Mitsch et al., 2015; Zedler & Kercher, 2005). Historically, human activities have exerted strong pressures on wetland ecosystems worldwide. This has resulted in significant impacts on these ecosystems (Davidson, 2014; Gilbert et al., 2017; Naveh & Lieberman, 1994), including biodiversity loss, increased eutrophication, and reduced water levels (Brinson & Malvárez, 2002; Dudgeon et al., 2006). Additionally, there is growing concern regarding the increasing presence of emerging contaminants (ECs) in wetlands (e.g., Celma et al., 2022; Lorenzo et al., 2019; Matamoros et al., 2012). ECs encompass diverse groups of chemicals, such as pharmaceuticals, pesticides, industrial chemicals, surfactants, endocrine-disrupting compounds, and microplastics, among others (Rosenfeld & Feng, 2011). These substances enter aquatic ecosystems through agricultural runoff, industrial effluents and, most notably, domestic wastewater. Municipal wastewater is treated in the best-case scenario in wastewater treatment facilities (WWTPs) (Overton et al., 2023). However, since conventional wastewater treatment technologies are not designed to remove ECs (Luo et al., 2014; Rizzo et al., 2019), a wide variety of pharmaceuticals and personal care products (PhPCPs), stimulants, illicit drugs, and pesticides are continuously released into the aquatic environment (Grandclément et al., 2017; Nuel et al., 2018; Yadav et al., 2017).

Eutrophication and EC threats are likely to be amplified in the context of climate change. For example, trophic conditions in water resources are affected by varying internal and external nutrient loadings driven by global temperature increases and changes in precipitation patterns, wind speed, and solar radiation intensity (Sharabian et al., 2018). As these authors recognized, maintaining our invaluable water resources in a changing climate is a major and significant challenge for policymakers today and deserves considerable attention for the sake of future generations. The impact of climate change is especially concerning for the Mediterranean basin, an area characterized by water scarcity. This region has been identified as one of the most vulnerable regions to the impacts of global climate change since the models used by the International Panel on Climate Change forecast predict an increase in average temperatures in the range of 2.2–5.1 °C for the period 2080–2100 (Pachauri et al., 2014). As a result, Mediterranean wetlands are expected to be highly influenced by climate change not only because of their geographic location but also because of their intrinsic features, such as temporality, shallowness, and typically high catchment area-to-lake area ratios (Álvarez-Cobelas et al., 2005; de Vicente, 2021; Parra et al., 2021).

In this context, there is an urgent need to develop techniques for reducing the impact of such stressors (eutrophication and ECs) in inland waters. Natural and constructed wetlands can both contribute to nutrient and contaminant attenuation, although they differ substantially in hydrology, design, management, and ecological function (Rahman et al., 2020). While eutrophication has historically been the primary focus (e.g., Ryding & Rast, 1989; Schindler, 1974; Vollenweider, 1968), the need to address EC fate in these systems is increasingly recognized. Mechanisms for EC removal within wetlands have been extensively studied (Matamoros et al., 2008; Verlicchi & Zambello, 2014) and comprehensively synthesized by Overton et al. (2023). These authors reported that the primary mechanisms discussed in the literature are sorption, photodegradation, microbial biodegradation, and phytoremediation. However, the most influential mechanism depends on the properties of the contaminant and wetland system studied. To date, constructed wetlands are considered a good alternative to conventional wastewater treatment systems, particularly in rural areas, because of their high removal efficiency of various contaminants, low energy consumption, modest operational requirements, and low maintenance costs (e.g., Wolff et al., 2024; Wu et al., 2015). It is also essential to take advantage of the purifying role of natural and constructed wetlands to increase the supply of water resources, as the human population is continuously growing, reaching 8 billion people (United Nations, 2022), all of whom need clean water (Weyhenmeyer et al., 2024). Moreover, constructed wetlands can mitigate the global decline in natural wetlands, a critical consideration in the Mediterranean region, where these ecosystems are particularly scarce. A potential concern is that the continuous input of ECs may lead to their accumulation within wetland food webs. However, the ecological consequences of such contamination are often difficult to detect using broad endpoints such as disease prevalence or short-term survival of higher trophic levels. For example, Ross et al. (2023) found no clear relationship between water pollution and disease prevalence or survival in migratory shorebirds. Nevertheless, this does not exclude the possibility of chronic or sublethal effects. ECs may alter physiological processes, induce endocrine disruption, or affect reproduction, behaviour, and species interactions. In addition, they may impact lower trophic levels, including zooplankton and aquatic invertebrates, which play key roles in ecosystem functioning and constitute important food resources for waterbirds.

Understanding the environmental degradation of ECs is crucial for optimizing the design and performance of artificial wetlands aimed at enhancing their removal efficiency. Recent studies emphasize that the effectiveness of constructed wetlands in removing ECs from wastewater is influenced primarily by three core components: the substrate media, vegetation, and the microbial community (Muduli et al., 2023). A comprehensive review by Overton et al. (2023) further highlighted that both biotic and abiotic removal mechanisms are largely determined by the physical–chemical properties of contaminants and intrinsic wetland characteristics such as hydrology, substrate composition, and vegetation structure. Additional strategies, such as water recirculation, have also been shown to significantly improve EC removal rates. In this context, Herrera-Melián et al. (2023) reported that full recirculation increased the EC removal efficiency from 87.5 to 97%, in addition to increasing ammonium and total nitrogen elimination. Moreover, the configuration of wetland systems plays a key role, with horizontal subsurface flow systems demonstrating greater efficiency than free water surface wetlands for the removal of nutrients and organic matter (Hernández-Crespo et al., 2017). Technical interventions, such as aeration or the installation of aeration pipes, may further increase EC removal despite associated costs (Martínez-Megías et al., 2024).

The present study was conducted in the Fuente de Piedra wetland (southern Spain). The main wetland basin receives treated wastewater routed through a series of semi-natural ponds whose hydrological connectivity changes markedly between the wet and dry seasons and, occasionally, during temporary bypass configurations put in place for restoration and vegetation-management works within the pond system. These contrasting circulation patterns provide a natural experiment to evaluate how different water-circulation regimes influence nutrient and specific ECs attenuation in a semi-natural pond system operating as a nature-based solution. An earlier study by de-los-Ríos-Mérida et al. (2017), which was based on a limited number of sampling days, reported notable reductions in nitrogen (N) and phosphorus (P) concentrations in this semi-natural pond system. However, no information exists regarding the system’s effectiveness in removing pesticides, stimulants, illicit drugs, and PhPCPs, despite previous research documenting high concentrations of compounds such as benzoylecgonine, cocaine, and naproxen in the groundwater of the surrounding area (Llamas-Dios et al., 2024; Vadillo et al., 2018).

In this context, we hypothesized that the attenuation of nutrients and ECs in the semi-natural pond system is strongly influenced by water-circulation regime and hydraulic residence time, with greater reduction occurring when treated wastewater flows through the semi-pond network before entering the Fuente de Piedra natural wetland. Accordingly, the objectives of this study were to (i) evaluate the role of the semi-natural pond system in reducing nutrient concentrations and attenuating selected pesticides, stimulants, illicit drugs, and PhPCPs before they reach the Fuente de Piedra main wetland basin, thereby mitigating environmental stress on the receiving ecosystem; (ii) assess changes in ecotoxicological risk along the system to quantify its effectiveness in reducing the potential ecological impacts of wastewater inputs; and (iii) determine the compound-specific behaviour of the selected pesticides, stimulants, illicit drugs, and PhPCPs under variable hydrological conditions.

## Materials and methods

### Study site

The Fuente de Piedra wetland (37°6′N, 4°44′W) is an endorheic, hypertrophic, and hypersaline system (13.5 km^2^; NE axis: 6.8 km; SE axis: 2.5 km) (García & Niell, 1993). It was the third Spanish wetland designated under the Ramsar convention (1983) and was declared a regional natural reserve in 1984. Its shallow water column provides ideal conditions for migrating and resident waterbirds, supporting one of the most important breeding colonies of the greater flamingo (*Phoenicopterus roseus* Pallas 1811) in the western Mediterranean (Rendón et al., 2014). Despite its protection status, one of the major pressures affecting this ecosystem is the discharge of treated wastewaters (de-los-Ríos-Mérida et al., 2017, 2021). The wetland receives effluents from three WWTPs. One of them, the Fuente de Piedra-II WWTP, is a pond-based treatment system consisting of three open-air stabilization ponds. The plant receives municipal wastewater from the town of Fuente de Piedra (approximately 3000 inhabitants), although wastewater inputs increase seasonally during holiday periods. Since the early 2000s, the effluent from Fuente de Piedra-II WWTP has been routed through a series of semi-natural ponds before reaching the Fuente de Piedra main wetland basin: Laguneto (27,801 m^2^, maximum depth of 1.28 m), Laguna de los Juncares (9222 m^2^, maximum depth of 0.39 m), and Los Juncares (19,828 m^2^, maximum depth of 0.20 m) (de-los-Ríos-Mérida et al., 2017, 2021). The semi-natural pond system is characterized by extensive helophytic vegetation, including *Bolboschoenus maritimus* (L.) Palla, *Juncus acutus* L., *Scirpoides holoschoenus* L. (Soják), *Phragmites australis* (Cav.) Steud., and *Tamarix* spp. Vegetation cover varies seasonally in response to water-level fluctuations and hydroperiod dynamics, which are characteristic of Mediterranean wetlands (Conde-Álvarez et al., 2012; García-Rodríguez et al., 2026). During temporary restoration and vegetation-management works within the semi-natural pond system (October–November 2023), treated wastewater flowed directly into the Fuente de Piedra main wetland basin through a bypass channel that avoided the semi-natural pond system. For the shake of clarity, the term semi-natural ponds refers to a series of interconnected shallow ponds comprising both natural and managed water bodies that receive treated wastewater from the Fuente de Piedra-II WWTP while retaining key natural wetland characteristics, including emergent vegetation, sediment-driven biogeochemical processes, seasonal hydrological variability, and ecological connectivity with the surrounding wetland.

### Sampling

**Fig. 1:**
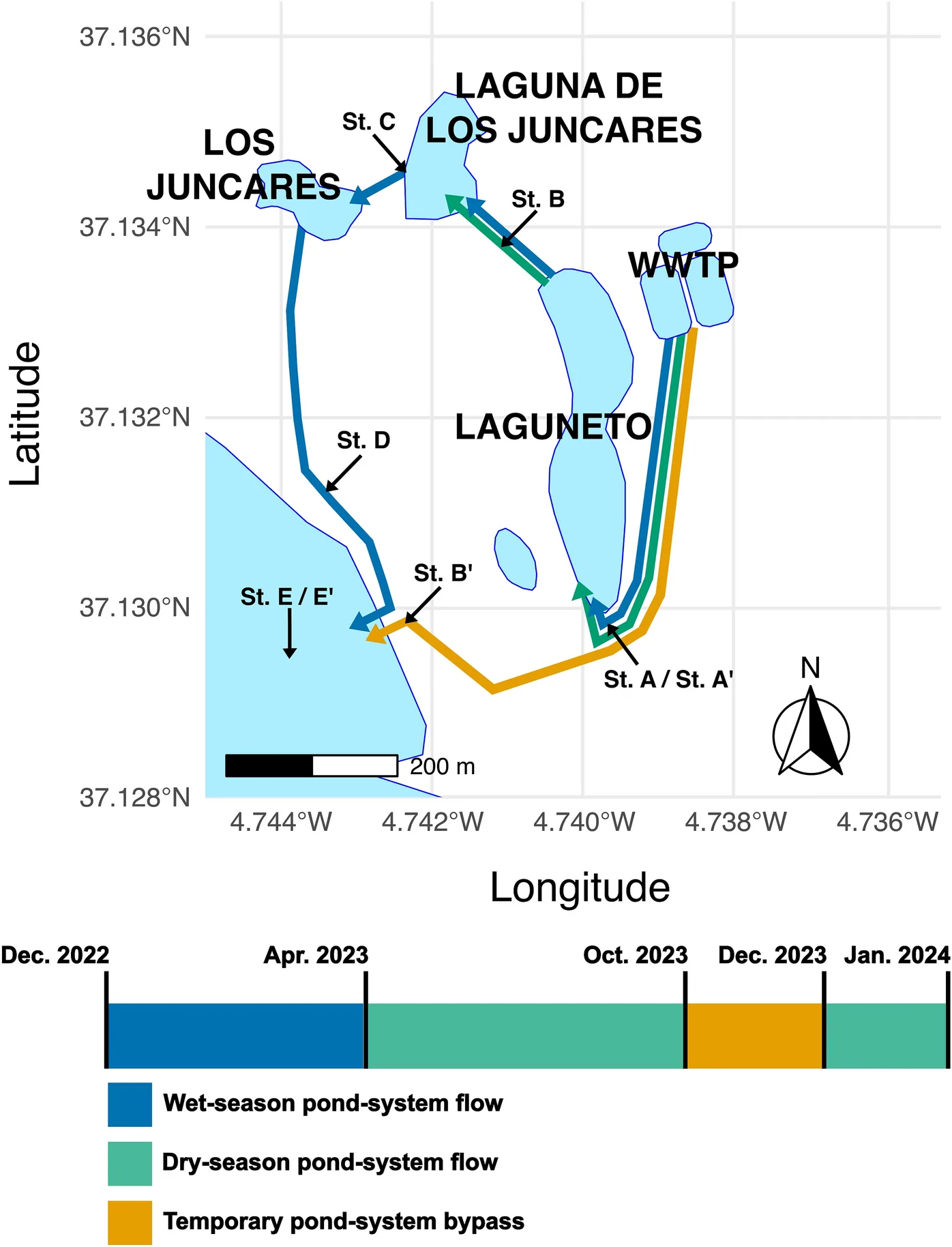
Water circulation scenarios and sampling design during the study period. The map shows the three water circulation scenarios from the wastewater treatment plant (WWTP) through the semi-natural pond system: (i) wet-season pond-system flow (blue line), with five sampling stations (A, B, C, D, and E); (ii) dry-season pond-system flow (green line), with three stations (A, B, and C); (iii) temporary pond-system bypass (orange line), with three alternative stations (A’, B’, and E’). Stations A and A’ and stations E and E’, correspond to the same physical locations. The timeline below the map indicates the implementation periods of each circulation scenario between December 2022 and January 2024

To assess water quality dynamics in the Fuente de Piedra wetland, 26 sampling events were conducted between December 2022 and January 2024 under three distinct hydrological scenarios (Fig. 1). Two scenarios corresponded to the regular WWTP effluent circulation through the semi-natural pond system before entering the Fuente de Piedra main wetland basin: the wet-season pond-system flow period (December 2022–April 2023), with five stations (A, B, C, D, and E), and the dry-season pond-system flow period (April 2023–October 2023 and December 2023–January 2024), with three stations (A, B, and C). Station A was located at the WWTP effluent channel, station B was in the Laguneto outlet area, station C was in Laguna de los Juncares downstream section, station D was in Los Juncares downstream section and station E was at Fuente de Piedra main wetland basin inlet. The third scenario corresponded to the temporary pond-system bypass period (October–November 2023) during restoration works in Laguna de los Juncares pond. Within this period, the flow path of the WWTP effluent was shortened and sampling was conducted at three stations (A′, B′, and E′), including the original stations A as A’ (WWTP effluent channel) and E as E’ (Fuente de Piedra main wetland basin inlet) and a new intermediate station (B′) located in the channel connecting them. These hydrological scenarios were defined according to the observed hydraulic connectivity and wastewater flow pathways within the pond system rather than solely according to seasonal climatic conditions. We estimated the hydraulic retention time (HRT) for the Laguneto pond (located between stations A and B) monthly as the ratio between the pond volume and the daily inflow from the WWTP. The pond volume was calculated from the Laguneto surface area and the monthly mean water depth, derived from daily field measurements with a staff gauge installed in the pond. The mean HRT was 46.7 (range: 26–62) days during the wet-season regular flow period and 45.3 (range: 40–55) days during the dry-season regular flow period. We also estimated the time required for the treated effluent to reach Fuente de Piedra main wetland basin from a wastewater treatment plant (WWTP) under the temporary bypass scenario. We calculated the hydraulic transit time (HTT) along the 850-m-long canal connecting both points. Based on the average daily discharge during the sampling period (634 m^3^ day^−1^) and assuming an open channel with an estimated width of 1.0 m and a water depth of 0.5 m (cross-sectional area of 0.5 m^2^), the mean flow velocity was estimated at 0.015 m s^−1^. Under these assumptions and considering plug flow conditions, the HTT was approximately 16 h, representing the time needed for the effluent to reach the Fuente de Piedra main wetland basin after discharge.

One sample was collected at each sampling station per sampling event. A representative composite sample was produced after combining two subsamples obtained from two locations within the station using a sampling pole. After homogenization, a 1500 mL polyethylene terephthalate (PET) bottle was filled for subsequent physical–chemical characterization, nutrient, and chlorophyll analyses. Water depth at the sampling locations generally did not exceed 50 cm. Therefore, subsamples were collected from the surface water layer, and efforts were made to maintain a similar sampling depth among stations. Water samples for EC determination were also collected in 250 mL amber PET bottles with Teflon caps. Water samples were transported from the field to the laboratory in a portable cooler at temperature below 4 °C. Transport time did not exceed 1.5 h. Upon arrival, samples were immediately filtered and stored at − 20 °C in darkness until analysis. Samples were typically analysed within 1–2 months of collection for both nutrient and ECs.

### Determination of physical–chemical, chemical and biological variables

The physical‒chemical parameters, including temperature, dissolved oxygen (DO), conductivity, and pH, were measured *in situ* via a YSI ProQuatro multiparameter probe. In the laboratory, total suspended solids (TSS) were determined by filtering a known volume through preweighed glass fibre filters (GFF, 0.7 µm pore size, Whatman) and then weighing the dried filter (at 105 °C for 24 h). Chlorophyll *a* (Chl *a*) was measured following Lorenzen (1967) and Ritchie (2006). Briefly, samples were filtered onto glass fibre filters, extracted in ethanol, and analysed spectrophotometrically with acidification to correct for phaeopigments. Water colour was measured at 440 nm and expressed as the absorption coefficient (*a*_*440*_, m⁻^1^) according to Reche and Pace (2002). Total dissolved iron (Tot-Fe_dis_) was quantified via the ferrozine method (Gibbs, 1979), and reactive silica (Si) was quantified via the ascorbic acid‒molybdate method (Koroleff, 1983). Dissolved inorganic phosphorus (DIP) was determined via the molybdenum blue method (Murphy & Riley, 1962). Total phosphorus (TP) and total dissolved phosphorus (TDP) were measured after potassium persulfate digestion of unfiltered and filtered aliquots, respectively (Rice et al., 2012). Total nitrogen (TN) and total dissolved nitrogen (TDN) were quantified by ultraviolet (UV) spectrophotometry after digestion (Rice et al., 2012). Dissolved inorganic nitrogen (DIN) species were determined individually: N–NH_4_⁺ by the phenate method (Rodier, 1981), N–NO_3_^−^ by UV screening (Rice et al., 2012), and N–NO_2_^−^ by the sulfanilamide method (Rodier, 1981). Major cations (Na⁺, K⁺, Mg^2^⁺, and Ca^2^⁺) and anions (SO_4_^2^⁻ and Cl⁻) were analysed via ion chromatography (IC; 940 Professional IC Vario, Metrohm).

### Emerging contaminant analysis

We selected 49 ECs belonging to various classes of pesticides, stimulants and illicit drugs, and PhPCPs. The list of targeted ECs, along with the liquid chromatography coupled with mass spectrometry (LC–MS) analytical conditions, is provided in Table S1 (supplementary information). The selection of ECs was based on a) wide application/use, b) amenability for (LC–MS) analysis, and c) occurrence previously reported in the Fuente de Piedra area (Llamas-Dios et al., 2024; Vadillo et al., 2018).

A volume of 500 mL was initially used for EC analysis. However, the sample volume was reduced to 100 mL in the following sample batches because of matrix interferences and the EC levels observed in the analysis. Before solid-phase extraction (SPE) of the ECs from water, a known amount (between 10 and 40 ng mL^−1^ in the final extract) of isotopically labelled compounds (listed in Table S1 as supplementary information) was added to the sample, which was subsequently filtered through glass fibre disks (47 mm, 1 µm pore size; Millipore, Merck). Then, ethylenediaminetetraacetic acid (EDTA) at a final proportion of 0.1% was added, and the pH was adjusted to 4.5 ± 0.3. Briefly, the filtrate was passed through Oasis HLB cartridges (200 mg and 60 mg used for the extraction of 500 mL and 100 mL of water, respectively, Waters Cromatografía, SA) previously conditioned with LC–MS-grade methanol (MeOH) and water adjusted to pH 4.5 (Labkem, Labbox Labware, S.L.). Upon sample loading, the cartridge was washed with water (5 mL in the case of 500 mL extraction or 2 mL in the case of 100 mL extraction) and dried under vacuum. The retained analytes were subsequently eluted with 2 × 3.5 mL MeOH. The eluate was dried under a gentle stream of nitrogen until dry, reconstituted with 1 mL of water:methanol (95:5) and filtered through 0.2 µM regenerated cellulose (RC) syringe filters.

For EC determination, the extracts (10 µL) were injected into a 1260 Infinity II liquid chromatograph interfaced with an electrospray source to a 6450 LC/TQ triple quadrupole analyser (Agilent). Analytes (listed in Table S1 as supplementary information) determined in positive ionization mode were separated via a Gemini column (2.3 µm, 2.1 × 100 mm, Phenomenex) and a linear gradient of methanol in an aqueous phase containing 0.1% formic acid (total analytical run time of 26 min). Analytes determined in negative ionization mode were separated with an XSelect HSS T3 column (2.5 µm, 2.1 × 100 mm, Waters) and eluted with a linear gradient of methanol in an aqueous phase containing 0.2 mM ammonium fluoride (total analytical run time of 24 min).

Mass acquisition was conducted with 4–5 min retention time windows in the selected reaction monitoring (SRM) mode by registering 2 SRMs per analyte and 1 SRM per isotopically labelled compound. The total analysis cycle time was 750 ms. Positive identification of the analyte was based on its retention time and the SRM1/SRM2 signal ratio, which must fit with those observed in an aqueous standard solution. Mass acquisition and data handling were performed with MassHunter Workstation (Agilent). Additional details on the LC–MS/MS method used and the methods used are provided in the supplementary material (Table S2 and S3 in the supplementary information).

### Ecotoxicity assessment

To determine spatial changes (from the outlet of the WWTP to the Fuente de Piedra main wetland basin) in EC-associated environmental risk, the risk quotient (RQ) method was applied at each sampling station during the wet-season pond-system flow and temporary pond-system bypass periods, in accordance with European Chemicals Agency guidelines (ECHA, 2016). The RQ for each compound was calculated as the ratio between its measured environmental concentration (MEC) and its predicted no-effect concentration (PNEC) for freshwater organisms via the following equation:

$${\mathrm{RQ}} = {\mathrm{MEC}}/{\mathrm{PNEC}}_{{{\mathrm{freshwater}}}}$$

The MECs used corresponded to either maximum concentrations (for worst-case scenario estimates) or mean concentrations (for average exposure assessment) at each sampling station. Only compounds with concentrations above the limit of quantification (LOQ) were included in the analysis, in line with European Union (EU) recommendations (EU, 2009).

PNEC values were obtained from the NORMAN ecotoxicology database (NORMAN, 2025), and the lowest available predicted or experimental toxicity value for freshwater organisms was selected to ensure conservative (worst-case) risk estimation. A comparison between the analytical method sensitivity and PNEC values has been provided in Table S4. The LOQ was below the corresponding PNEC for all compounds, except for imidacloprid, whose LOQ and PNEC present similar values (6.8 ng/L vs. 5–10 ng/L). Thus, the method used presents an adequate analytical sensitivity for correct ecotoxicological assessment with the RQ approach.

ECs were categorized into four risk levels according to their individual RQ values: RQ < 0.1 (no risk), 0.1 < RQ < 1 (low risk), 1 < RQ < 10 (moderate risk), and RQ > 10 (high risk). To estimate the overall risk at each sampling station and date, a cumulative risk quotient (RQ_sample_) was calculated by summing the RQs of all ECs detected above the LOQ in each sample (Barbieri et al., 2021; Postigo et al., 2023). This additive model provides an estimate of the combined risk posed by chemical mixtures, although it assumes similar modes of action and does not account for potential synergistic or antagonistic effects.

### Statistical analyses

All the statistical analyses were performed via R software (R Core Team, 2025). For each variable (physical–chemical parameters, nutrients, Chl *a*, and individual ECs), differences among the sampling stations were assessed independently for each water-circulation scenario: wet-season pond-system flow, dry-season pond-system flow, and temporary pond-system bypass. For each scenario, the Friedman test was used to detect overall differences among stations, as the data did not meet the assumptions of parametric repeated-measures ANOVA. When the Friedman test was significant (*P*-value < 0.05), we performed paired comparisons between stations. Normality was evaluated with the Shapiro–Wilk test applied to the paired differences (not to the raw data) (McDonald, 2014). If these differences were normally distributed (*P*-value > 0.05), we used paired t-tests; otherwise, we used Wilcoxon signed-rank tests. To correct for multiple testing, *P*-values from all pairwise comparisons were adjusted via the Benjamini–Hochberg procedure to control the false discovery rate (FDR; Benjamini & Hochberg, 1995). Adjusted *P*-values (*Q*-values) less than 0.05 were considered statistically significant.

## Results

### Effects of water circulation on physical–chemical, chemical and biological variables

Water temperature across the semi-natural pond system and the main basin of the Fuente de Piedra wetland ranged from 7.8 to 28.2 °C in the wet-season pond-flow period and from 5.6 to 26.6 °C in the dry-season pond-flow period (Table 1). Temperatures during the temporary bypass period varied between 12.9 and 22.9 °C. DO increased along the flow path from the WWTP effluent channel (station A) towards the Fuente de Piedra main wetland basin inlet (station E) during the wet-season pond-flow period, showing a marginally significant trend (*P*-value = 0.036; *Q*-value = 0.084). Similar increases were observed during the dry-season pond-flow period along the pond network (stations A–C) and during the temporary bypass period between the WWTP effluent channel and the main wetland basin inlet (stations A′–E′), with significant differences supported by *Q-*values of 0.016 and 0.020, respectively. Conductivity also varied significantly along the wet-season pond-flow pathway (*P*-value = 0.036; *Q*-value = 0.080). Mean pH values during the wet-season pond-flow period ranged from 7.11 to 9.73. During the dry-season pond-flow period, pH decreases significantly between stations A and C, with the highest recorded value (9.14) recorded at station B. Colour (*a*_440_) declined progressively as water moved through the pond network towards the main wetland basin during the wet-season pond-flow period, with a significant reduction of 64% between station A and E. In contrast, TSS did not differ significantly among sampling stations under any hydrological scenario (*Q-*value > 0.05). Mean Tot-Fe_dis_ concentrations ranged from 1 µg L^−1^ at station B during the wet-season pond-flow period to 151 µg L^−1^ at station C during the dry-season pond-flow period, with concentrations exceeding 20 µg L^−1^ at stations E, C and E’ during all three under the three hydrological scenarios (Table 1). During the wet-season pond-flow period, Si concentrations decreased significantly along the flow pathway from the WWTP discharge point (station A) to the Fuente de Piedra main wetland basin (station E) (*P*-value = 0.025; *Q*-value = 0.054). In general, higher Tot-Fe_dis_ and Si concentrations were recorded the dry-season pond-flow period than during the other hydrological scenarios.

**Table 1:** Mean values ± SD at the sampling stations during the wet- and dry-season pond-system flows, and the temporary pond-system bypass for selected water physicochemical variables

Wet-season pond-system flow						
	Station A	Station B	Station C	Station D	Station E	% red. A-E
n	6	6	6	6	6	
T	13.40 ± 3.08^a^	15.78 ± 6.24^a^	16.03 ± 5.35^a^	15.82 ± 3.22^a^	17.13 ± 6.47^a^	–
DO	3.59 ± 3.11^a^	6.70 ± 3.57^a^	9.85 ± 4.96^a^	6.34 ± 5.29^a^	6.71 ± 3.84^a^	–
Cond	3.08 ± 0.46^a^	2.99 ± 0.38^a^	2.85 ± 0.29^a^	4.67 ± 1.99^a^	14.89 ± 17.78^a^	–
pH	8.20 ± 0.10^a^	8.38 ± 0.28^a^	8.80 ± 0.41^a^	8.13 ± 0.76^a^	8.27 ± 0.90^a^	–
*a*_440_	11.98 ± 3.26^a^	11.02 ± 6.29^abc^	7.49 ± 3.18^abc^	6.45 ± 0.76^b^	4.23 ± 1.80^c^*	64*
TSS	50.17 ± 22.62^a^	61.17 ± 40.53^a^	161.17 ± 62.65^a^	63.67 ± 55.75^a^	91.83 ± 64.16^a^	–
Tot-Fe_dis_	7.50 ± 3.78^a^	0.33 ± 052^a^	ND	0.83 ± 1.33^a^	20.83 ± 26.69^a^	–
Si	2.05 ± 0.66^a^	1.50 ± 0.68^b^	0.77 ± 0.39^b^	0.51 ± 0.40^b^	0.97 ± 0.80^ab^	52

Dry-season pond-system flow					Temporary pond-system bypass			
	Station A	Station B	Station C	% red. A-C	Station A’	Station B’	Station E’	% red. A’-E’
n	15	15	10		4	4	4	
T	19.05 ± 2.30^a^	19.46 ± 2.54^a^	18.66 ± 3.95^a^	2	16.70 ± 3.27^a^	16.60 ± 3.32^a^	17.28 ± 4.32^a^	–
DO	3.62 ± 1.39^a^	5.59 ± 3.04^b^	5.40 ± 3.22^b^	–	1.96 ± 0.50^a^	2.03 ± 0.56^a^	7.03 ± 1.79^b^	–
Cond	4.29 ± 0.42^a^	4.46 ± 0.14^b^	3.16 ± 0.48^b^	26*	1.70 ± 0.69^a^	1.81 ± 0.45^a^	1.90 ± 0.50^a^	–
pH	9.04 ± 0.12^a^	9.45 ± 0.49^b^	8.38 ± 0.54^b^	7*	8.12 ± 0.17^a^	8.12 ± 0.06^a^	8.38 ± 0.12^b^	–
*a*_440_	11.39 ± 6.56^a^	9.31 ± 4.91^a^	7.97 ± 4.61^a^	37	3.80 ± 0.55^a^	4.49 ± 0.85^a^	3.22 ± 0.80^a^	15
TSS	66.24 ± 16.23^a^	95.09 ± 39.76^a^	122.27 ± 64.15^a^	–	54.00 ± 23.22^a^	39.75 ± 13.60^a^	55.75 ± 49.65^a^	–
Tot-Fe_dis_	23.20 ± 16.31^a^	11.80 ± 15.09^a^	23.73 ± 45.94^a^	–	20.48 ± 4.49^a^	44.06 ± 24.62^a^	31.40 ± 13.74^a^	–
Si	11.49 ± 3.30^a^	8.35 ± 3.08^a^	6.68 ± 4.18^a^	41	7.00 ± 1.86^a^	9.23 ± 8.53^a^	8.83 ± 7.71^a^	–

*T* temperature (°C); *DO* dissolved oxygen (mg L^-1^); *Cond* conductivity (mS cm^-1^); *a*_440_: colour (m^-1^); *TSS* total suspended solids (mg L^-1^); *Tot-Fe*_*dis*_ total dissolved iron (µg L^-1^); *Si* reactive silica (mg L^-1^)

Different lowercase letters indicate significant differences between sampling stations (*Q*-value < 0.05). *ND* not detected. The percentage reductions (% red.) between stations A–E, A–C and A’–E’ that were significant are marked with an asterisk (*) (*Q*-value < 0.05). (-) indicates an increase

**Fig. 2:**
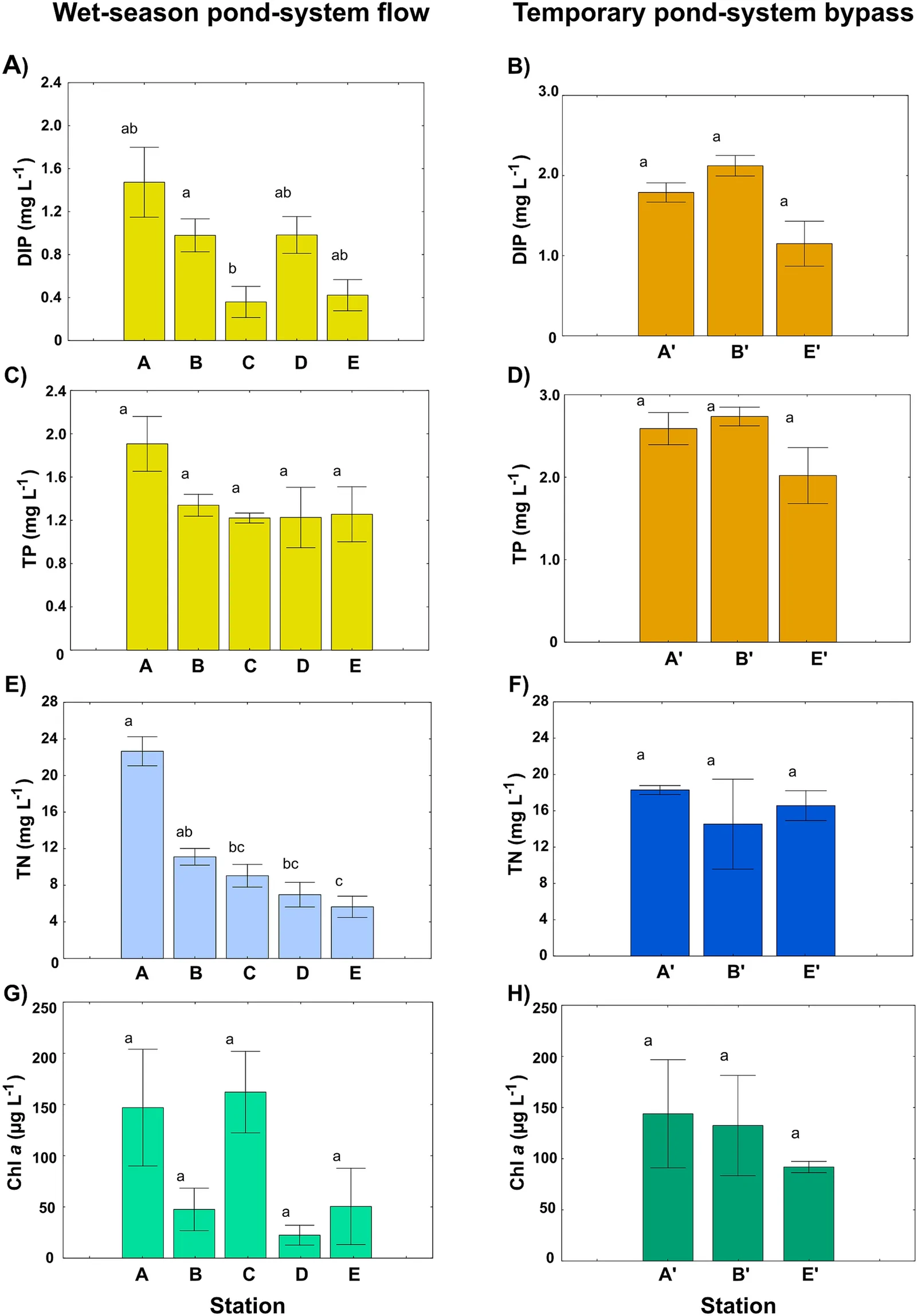
Spatial variation in the concentrations of dissolved inorganic phosphorus (DIP), total phosphorus (TP), total nitrogen (TN), and chlorophyll a (Chl *a*) during the wet-season pond-system flow (December 2022–April 2023) and the modified water circulation period (temporary pond-system bypass: October–November 2023). Bars represent standard errors (± SE). Different lowercase letters indicate statistically significant differences between sampling stations (*Q*-value < 0.05)

DIP exhibited contrasting spatial patterns among hydrological scenarios (Table 2). During the wet-season pond-flow period, DIP concentrations declined progressively as treated wastewater moved through the semi-natural pond network towards the main wetland basin, decreasing by approximately 60% between stations A and E (Fig. 2A; P = 0.042; Q = 0.084). An even stronger reduction was observed during the dry-season pond-flow period, with DIP concentrations decreasing by 80% between stations A and C (Table 1). In contrast, DIP concentrations remained relatively constant between the WWTP effluent channel and the main wetland basin during the temporary bypass period, when wastewater bypassed most of the pond system (Fig. 2B).

**Table 2:** Mean values ± SD at the sampling stations during the wet- and dry-season pond-system flows, and the temporary pond-system bypass for selected nutrient concentrations and chlorophyll *a*

Wet-season pond-system flow						
	Station A	Station B	Station C	Station D	Station E	% red. A-E
n	6	6	6	6	6	
DIP	1.18 ± 0.42^ab^	1.09 ± 0.32^a^	0.41 ± 0.39^b^	0.98 ± 0.42^ab^	0.48 ± 0.39^ab^	59
TDP	1.50 ± 0.43^a^	1.09 ± 0.35^ab^	0.43 ± 0.30^c^	0.92 ± 0.45^bc^	0.69 ± 0.30^abc^	53
TP	1.69 ± 0.39^a^	1.36 ± 0.29^a^	1.25 ± 0.10^a^	1.23 ± 0.68^a^	1.32 ± 0.71^a^	21
N–NO_3_^−^	4.59 ± 4.93^a^	1.27 ± 0.93^a^	0.37 ± 0.17^a^	0.29 ± 0.17^a^	0.41 ± 0.13^a^	91
N–NO_2_^−^	1.06 ± 0.86^a^	0.64 ± 0.65^a^	0.11 ± 0.06^a^	0.02 ± 0.03^a^	0.03 ± 0.02^a^	97
N–NH_4_^+^	14.15 ± 4.76^a^	7.60 ± 2.80^ab^	3.64 ± 4.40^c^	3.51 ± 4.05^bc^	2.41 ± 1.88^c^	82*
DIN	19.80 ± 8.81^a^	9.51 ± 2.52^a^	2.31 ± 2.84^b^	3.82 ± 4.20^b^	2.85 ± 1.91^b^	85*
TN	22.53 ± 4.59^a^	11.14 ± 2.64^ab^	8.95 ± 3.57^bc^	6.97 ± 3.30^bc^	5.5 ± 3.36^c^	75*
Chl *a*	146.96 ± 139.70^a^	47.55 ± 50.98^a^	162.12 ± 97.4^a^	22.41 ± 23.82^a^	50.48 ± 91.23^a^	65

Dry-season pond-system flow					Temporary pond-system bypass			
	Station A	Station B	Station C	% red. A-C	Station A’	Station B’	Station E’	% red. A’-E’
n	15	15	10		4	4	4	
DIP	1.92 ± 0.75^a^	0.77 ± 0.45^b^	0.40 ± 0.25^c^	79*	1.79 ± 0.27^a^	2.12 ± 0.26^a^	1.15 ± 0.43^a^	23
TDP	1.96 ± 0.55^a^	1.33 ± 0.61^a^	0.71 ± 0.27^b^	63*	1.94 ± 0.78^a^	2.60 ± 0.22^a^	1.77 ± 0.55^a^	11
TP	2.72 ± 0.72^a^	1.75 ± 0.37^b^	1.45 ± 0.46^b^	46*	2.58 ± 0.43^a^	2.73 ± 0.22^a^	2.02 ± 0.36^a^	0
N–NO_3_^−^	1.15 ± 1.76^a^	0.59 ± 0.35^a^	0.54 ± 0.20^a^	52	0.79 ± 1.15^a^	0.13 ± 0.12^a^	0.18 ± 0.49^a^	58
N–NO_2_^−^	0.35 ± 1.06^a^	0.18 ± 0.17^a^	0.06 ± 0.11^b^	82*	0.21 ± 0.23^a^	0.01 ± 0.01^a^	0.01 ± 0.08^a^	92
N–NH_4_^+^	17.65 ± 3.83^a^	4.28 ± 2.07^b^	2.46 ± 1.39^c^	86*	11.45 ± 1.69^a^	12.66 ± 2.14^a^	11.60 ± 5.00^a^	0
DIN	19.15 ± 7.51^a^	5.04 ± 3.27^b^	3.07 ± 2.06^c^	83*	12.34 ± 1.84^a^	14.27 ± 2.85^a^	10.65 ± 4.04^a^	13
TN	20.49 ± 2.28^a^	12.92 ± 4.61^b^	11.35 ± 1.59^b^	44*	18.29 ± 1.09^a^	14.53 ± 9.90^a^	16.57 ± 2.92^a^	2
Chl *a*	125.48 ± 89.70^a^	311.12 ± 244.03^a^	169.43 ± 112.72^a^	–	137.00 ± 135.35^a^	132.48 ± 98.04^a^	93.04 ± 10.93^a^	32

*DIP* dissolved inorganic phosphorus (mg L^-1^); *TDP* total dissolved phosphorus (mg L^-1^); *TP* total phosphorus (mg L^-1^); N–NO_3_^-^ (mg L^-1^); N–NO_2_ (mg L^−1^); N–NH_4_^+^ (mg L^-1^); *DIN* total dissolved nitrogen (mg L^-1^); *TN* total nitrogen (mg L^-1^); Chl *a*: chlorophyll *a* (µg L^-1^).

Different lowercase letters indicate significant differences between sampling stations (*Q*-value < 0.05). *ND* not detected. The percentage reductions (% red.) between stations A–E, A–C and A’–E’ that were significant are marked with an asterisk (*) (*Q*-value < 0.05). (-) indicates an increase.

TDP also declined along the semi-natural pond network during both pond-flow scenarios. During the wet-season pond-flow period, mean TDP concentrations decreased significantly from the WWTP effluent channel (station A) towards the downstream stations, resulting in an overall reduction of 53% between stations A and E (*P*-value = 0.033; *Q*-value = 0.083). Similar reductions were observed during the dry-season pond-flow period between stations A and C. In contrast, TP exhibited different spatial patterns depending on the hydrological scenario. During the wet-season pond-flow period, TP concentrations remained relatively similar among stations (Fig. 2C), whereas during the dry-season pond-flow period the highest mean concentrations was recorded at station A and decreased significantly downstream. During the temporary bypass period, TP concentrations showed no significant spatial variation and remained consistently high (> 2 mg L⁻^1^), reaching a maximum of 3 mg L⁻^1^ (Fig. 2D). Dissolved inorganic N species (N–NO_3_^−^, N–NO_2_^−^, and N–NH_4_⁺) exhibited marked reductions as treated wastewater moved through the semi-natural pond system during both pond-flow scenarios. During the wet-season pond-flow period, N–NO_3_^−^, N–NO_2_^−^, and N–NH_4_⁺ concentrations between the WWTP effluent channel (station A) and the main wetland basin (station E), with the strongest reductions observed for N–NO_2_^−^ (97%; P-value = 0.031; Q-value = 0.090) and N–NH_4_⁺ (82%; Q-value = 0.012). Similar downstream decreases in N–NO_2_^−^ and N–NH_4_⁺ were observed the dry-season pond-flow period between stations A and C. Consequently, DIN concentrations declined significantly under both pond-flow scenarios. A comparable pattern was observed for TN (Fig. 2E), which also decreased significantly along the pond network during both seasons. In contrast, during the temporary bypass period (Fig. 2F), DIN concentrations remained consistently high (> 10 mg N L^−1^) and showed little spatial variation between the WWTP effluent channel and the Fuente de Piedra main wetland basin. Likewise, TN concentrations did not vary significantly under bypass conditions.

Chl *a* concentrations showed high spatial variability under all hydrological scenarios (Table 1). During the wet-season pond-flow period, concentrations ranged from 1.10 at the main wetland basin (station E) to 402.23 µg L^−1^ at the WWTP effluent channel (station A), although no significant spatial differences were detected (Fig. 2G). The highest concentrations observed during the study (930.50 µg L^−1^) was recorded at station B during the dry-season pond-flow period. During the temporary bypass period, Chl *a* concentrations ranged from 35.07 (station B’) to 337.57 µg L^−1^ (station A’) (Fig. 2H).

Under all hydrological scenarios, Cl⁻ was the dominant ion, followed by SO_4_^2^⁻ and Na⁺ (Table 3). During the wet-season pond-flow period, Ca^2^⁺ concentrations decreased along the flow pathway from station A to main wetland basin (station E), whereas Mg^2^⁺ increased. Na⁺ (*P*-value = 0.027; *Q*-value = 0.069), K⁺ (*P*-value = 0.036; *Q*-value = 0.061) and Cl⁻ (*P*-value = 0.036; *Q*-value = 0.102) also increased towards the downstream stations. Similar trends were observed during the dry-season pond-flow period, with significant increases in Na⁺, K⁺, Mg^2^⁺, Cl⁻, and SO_4_^2^⁻ between stations A and C, while Ca^2^⁺ showed no significant spatial variation. During the temporary bypass period Na⁺ and Mg^2^ also increased between the most distant stations (A' and E').

**Table 3:** Mean values ± SD during the three sampling periods for major cations and anions (ppm) at the sampling stations

Wet-season pond-system flow					
	Station A	Station B	Station C	Station D	Station E
n	6	6	6	6	6
Na^+^	342.03 ± 31.58^a^	354.75 ± 54.95^a^	375.09 ± 65.26^a^	690.23 ± 304.64^a^	802.31 ± 294.87^a^
K^+^	10.49 ± 1.29^a^	12.30 ± 1.06^a^	12.58 ± 1.25^a^	15.59 ± 3.47^a^	20.71 ± 0.96^a^
Mg^2+^	45.95 ± 3.98^a^	48.28 ± 7.22^a^	49.83 ± 7.29^a^	96.36 ± 40.20^b^	125.99 ± 36.89^b^
Ca^2+^	301.12 ± 30.55^a^	242.05 ± 41.90^b^	171.59 ± 37.48^c^	195.60 ± 40.56^bc^	188.13 ± 31.31^bc^
Cl^−^	899.37 ± 133.66^a^	927.80 ± 225.70^a^	901.20 ± 180.07^a^	1685.92 ± 879.51^a^	5424.68 ± 6608.78^a^
SO_4_^2−^	274.16 ± 36.62^a^	278.07 ± 64.31^a^	304.00 ± 73.85^a^	335.70 ± 98.49^a^	914.17 ± 1257.94^a^

Dry-season pond-system flow			
	Station A	Station B	Station C
n	15	15	10
Na^+^	316.35 ± 88.19^a^	432.01 ± 68.49^b^	479.08 ± 146.15^c^
K^+^	11.99 ± 1.99^a^	15.55 ± 2.45^b^	16.88 ± 4.16^c^
Mg^2+^	42.91 ± 11.11^a^	55.32 ± 7.59^b^	58.50 ± 10.98^c^
Ca^2+^	195.19 ± 67.38^a^	188.61 ± 39.59^a^	185.83 ± 53.72^a^
Cl^−^	676.42 ± 185.20^a^	946.40 ± 111.93^b^	1104.31 ± 335.31^c^
SO_4_^2−^	212.58 ± 62.18^a^	276.12 ± 61.80^b^	306.04 ± 128.09^c^

Temporary pond-system bypass			
	Station A’	Station B’	Station E’
n	4	4	4
Na^+^	271.29 ± 97.33^ab^	340.81 ± 21.40^a^	321.12 ± 17.20^b^
K^+^	11.05 ± 1.98^a^	12.18 ± 0.31^a^	11.86 ± 0.92^a^
Mg^2+^	39.10 ± 14.34^ab^	52.05 ± 4.29^a^	46.97 ± 2.80^b^
Ca^2+^	126.52 ± 44.68^a^	134.63 ± 18.95^a^	134.21 ± 12.34^a^
Cl^−^	554.44 ± 198.84^a^	689.55 ± 56.74^a^	699.52 ± 38.58^a^
SO_4_^2−^	188.87 ± 66.68^a^	214.97 ± 17.59^a^	223.97 ± 19.71^a^

Na^+^sodium; K^+^: potassium; Mg^2+^: magnesium; Ca^2+^: calcium; Cl^−^: chloride; SO_4_^2−^: sulfate. Different lowercase letters indicate significant differences between sampling stations (*Q*- alue < 0.05)

### Effects of the water circulation system on emerging contaminant occurrence

In total, 33 out of the 49 ECs analysed were detected in at least one of the water samples collected during the study period (Table S1).

#### Pesticides

During the three hydrological scenarios, 13 pesticides were quantified (Table 4). All detected pesticides were present at all sampling stations, except dimethoate, which was not detected at station E during the wet-season pond-flow period nor at stations B’ and E’ during the temporary bypass period, and diazinon, which was not detected at station A’ during the temporary bypass period (Figure S1). Neonicotinoid insecticides were the dominant pesticide group, with acetamiprid and imidacloprid showing the highest concentrations during both pond-flow scenarios. Acetamiprid remained elevated from the WWTP effluent channel (station A) through the upstream ponds, whereas imidacloprid was primarily concentrated at the effluent channel. Other frequently detected pesticides included dimethoate, diazinon, terbuthylazine-desethyl, terbuthylazine, carbendazim, and the atrazine metabolites desethyl-atrazine and desisopropyl-atrazine, all of which exhibited mean concentrations exceeding 30 ng L⁻^1^ during the study period. During the wet-season pond-flow period, several triazine herbicides including atrazine, desethylatrazine, simazine, and terbutylazine-desethyl, showed significant concentration declines along the flow pathway from the WWTP effluent channel to the main wetland basin, with removal efficiencies exceeding 75%. Similar downstream decreases were observed for carbendazim and diuron, with mean reductions of 67% and 48%, respectively. During the dry-season pond-flow period, atrazine, carbendazim, diuron, diazinon, simazine, tebuconazole, terbuthylazine and terbuthylazine-desethyl also decreased significantly as water moved through the pond network, although removal efficiencies were generally lower than those observed during the wet-season pond-flow period. In contrast, pesticide concentrations showed little spatial variation during the temporary bypass period, with no significant differences detected between the WWTP effluent channel (station A′) and the main wetland basin (station E′).

**Table 4:** Mean concentrations (± SD; ng L⁻^1^) of quantified pesticides at the sampling stations during the three hydrological scenarios. Different lowercase letters indicate significant differences between sampling stations (*Q*-value < 0.05)

Wet-season pond-system flow						
	Station A	Station B	Station C	Station D	Station E	% red. St. A-E
n	6	6	6	6	6	
ACE	7.66 ± 5.66^a^	5.11 ± 1.57^a^	6.57 ± 0.86^a^	3.63 ± 1.27^a^	7.07 ± 4.11^a^	7
ATZ	9.66 ± 2.53^a^	6.16 ± 3.20^ab^	6.46 ± 1.23^a^	3.34 ± 0.90^b^	2.38 ± 0.70^b^	75*
CARB	33.07 ± 10.99^ab^	26.17 ± 5.19^ab^	33.28 ± 14.07^ac^	16.84 ± 8.50^bd^	10.87 ± 6.79^ cd^	67*
DEA	32.89 ± 8.34^a^	18.52 ± 7.68^bc^	19.96 ± 4.43^b^	12.47 ± 4.16^c^	6.06 ± 2.14^d^	81*
DIA	33.76 ± 15.33^a^	18.68 ± 10.84^a^	14.70 ± 6.62^a^	8.75^†^	6.69^†^	80
DIAZ	37.61 ± 83.94^a^	23.93 ± 27.23^a^	55.50 ± 54.03^a^	31.51 ± 27.33^a^	4.73 ± 4.70^a^	87
DIU	17.65 ± 5.84^a^	13.98 ± 5.02^abc^	15.46 ± 7.65^ab^	9.23 ± 4.82^c^	9.06 ± 2.17^bc^	48*
DMT	4.84^†^	3.30^†^	8.38^†^	5.17^†^	ND	100
IMI	122.66 ± 51.84^a^	40.92 ± 14.42^a^	39.08 ± 23.92^a^	37.74 ± 21.37^a^	21.28 ± 6.40^a^	82
SMZ	9.68 ± 2.73^a^	5.99 ± 2.96^ab^	6.35 ± 1.75^a^	2.81 ± 1.13^b^	1.94 ± 0.56^b^	80*
TBZ	5.92 ± 1.57^a^	2.59 ± 1.15^ab^	3.55 ± 2.41^ab^	1.47 ± 0.68^b^	2.85 ± 4.26^ab^	51
TEB	37.88 ± 33.65^a^	19.77 ± 13.26^a^	15.72 ± 5.45^a^	16.72 ± 20.59^a^	11.58 ± 5.61^a^	69
TERBDES	55.11 ± 13.45^a^	28.70 ± 11.24^b^	30.29 ± 5.97^b^	16.53 ± 5.60^c^	8.32 ± 4.87^d^	84*

Dry-season pond-system flow				% red. St. A-C	Temporary pond-system bypass			
	Station A	Station B	Station C		Station A’	Station B’	Station E’	% red. St. A’-E’
n	15	15	10		4	4	4	
ACE	172.97 ± 309.55^a^	168.66 ± 170.47^a^	159.85 ± 151.91^a^	7	26.17 ± 20.41^a^	24.99 ± 18.27^a^	24.90 ± 18.73^a^	4
ATZ	3.14 ± 2.10^a^	2.54 ± 1.83^a^	2.50 ± 1.45^b^	20*	1.40 ± 0.54^a^	1.63 ± 0.13^a^	1.61 ± 0.17^a^	–
CARB	15.69 ± 9.89^a^	12.66 ± 7.30^ab^	14.27 ± 7.26^b^	9*	10.38 ± 4.27^a^	9.84 ± 3.33^a^	9.52 ± 3.00^a^	8
DEA	11.87 ± 7.63^a^	10.45 ± 5.88^a^	11.65 ± 5.92^a^	1	5.22 ± 2.15^a^	5.88 ± 0.43^a^	5.78 ± 0.67^a^	–
DIA	19.27 ± 5.65^†^	20.39 ± 6.73^†^	15.68^†^	18	ND	5.80 ± 0.14^a^	5.87 ± 0.30^a^	–
DIAZ	1.01 ± 0.70^a^	0.57 ± 0.49^b^	0.54 ± 0.34^b^	46*	0.83 ± 0.31^a^	0.64 ± 0.19^a^	0.66 ± 0.18^a^	21
DIU	14.37 ± 4.96^a^	7.33 ± 1.67^b^	5.39 ± 1.26^c^	62*	11.73 ± 6.24^a^	9.90 ± 2.88^a^	10.43 ± 2.66^a^	11
DMT	44.12 ± 38.68^a^	58.54 ± 41.48^a^	40.05 ± 19.70^a^	9	5.37 ± 0.02^†^	ND	ND	100
IMI	99.00 ± 125.19^a^	27.61 ± 27.90^a^	24.88 ± 23.95^a^	74	25.51 ± 8.39^a^	21.66 ± 8.39^a^	23.79 ± 4.73^a^	6
SMZ	4.68 ± 2.57^a^	3.63 ± 2.45^a^	3.41 ± 2.06^b^	27*	2.68 ± 1.23^a^	3.12 ± 0.42^a^	3.08 ± 0.40^a^	–
TBZ	2.11 ± 0.95^a^	1.38 ± 1.03^b^	1.06 ± 0.62^c^	49*	1.40 ± 0.47^a^	1.53 ± 0.07^a^	1.45 ± 0.12^a^	–
TEB	8.59 ± 7.85^a^	5.83 ± 5.33^b^	6.62 ± 3.84^b^	22*	2.84 ± 1.57^a^	2.42 ± 0.50^a^	2.56 ± 0.44^a^	9
TERBDES	18.55 ± 15.85^a^	14.46 ± 11.57^b^	16.79 ± 10.81^c^	9*	5.46 ± 2.13^a^	6.39 ± 0.59^a^	6.22 ± 0.68^a^	–

*ACE* Acetamiprid; *ATZ* Atrazine; *CARB* Carbendazim; *DEA* Desethyl-atrazine; *DIA* Desisopropyl-atrazine; *DIAZ* Diazinon; *DIU* Diuron; *DMT* Dimethoate; *IMI* Imidacloprid; *SMZ* Simazine; *TBZ* Terbuthylazine; *TEB* Tebuconazole; *TERBDES* Terbuthylazine-desethyl

Significant reductions are indicated by an asterisk (*). *ND* not detected; † statistical comparison not performed (n < 3)

#### Stimulants and illicit drugs

Seven stimulants and illicit drugs were quantified during the wet-season pond-flow period (Table 5). Among stimulants, caffeine showed the highest mean concentration (6.67 µg L^−1^), followed by cotinine (1.15 µg L^−1^), with detection frequencies of 100% and above 16%, respectively. Among illicit drugs, benzoylecgonine and cocaine exhibited the highest mean concentrations (787 and 127 and ng L^−1^, respectively). Benzoylecgonine was the most abundant illicit drugs during the study and was present at all stations and sampling events. MDMA, ketamine, and morphine were also frequently detected at the WWTP effluent channel (station A) (detection frequency > 80%), with morphine showing the highest concentrations among these compounds. During the wet-season pond-flow period concentrations of several compounds decreased markedly as treated wastewater moved through the semi-natural pond network towards the main wetland basin. Caffeine exhibited the greatest reduction, decreasing by more than 90% between stations A and E (*P*-value = 0.038; *Q*-value = 0.104). During the dry-season pond-flow period, two additional compounds were detected: amphetamine and the cannabis metabolite 11-nor-Δ^9^-tetrahydrocannabinol-9-carboxylic acid (THC-COOH), the latter occurring exclusively at the WWTP effluent channel. Benzoylecgonine, caffeine and cocaine showed reductions exceeding 90%, along the pond network, while cotinine and MDMA decreased by more than 75%. In contrast, concentrations of stimulants and illicit drugs showed little spatial variation during the temporary bypass period, remaining relatively constant between the effluent channel (station A′) and the main wetland basin (station E′). Cocaine was the only compound exhibiting a significant decrease between stations A’ and E’. The heroin metabolite 6-monoacetylmorphine remained consistently below the method detection limit in all analysed samples.

**Table 5:** Mean concentrations (± SD; ng L⁻^1^) of quantified stimulants and illicit drugs at the sampling stations during the three hydrological scenarios. Different lowercase letters indicate significant differences between sampling stations (*Q*-value < 0.05)

Wet-season pond system flow						
	Station A	Station B	Station C	Station D	Station E	% red. St. A-E
n	6	6	6	6	6	
AM	ND	ND	ND	ND	ND	–
BE	786.94 ± 838.29^a^	180.96 ± 181.34^a^	324.27 ± 262.36^a^	122.53 ± 129.21^a^	487.36 ± 796.35^a^	38
CAF	6672.86 ± 5560.95^a^	745.87 ± 504.64^a^	571.32 ± 269.74^a^	323.46 ± 181.60^a^	462.47 ± 296.91^a^	93
COC	127.12 ± 155.37^a^	23.60 ± 25.16^a^	8.29 ± 9.59^a^	3.96 ± 4.51^a^	8.64 ± 7.32^a^	93
COT	1147.50 ± 747.87^a^	112.09 ± 85.26^a^	56.23 ± 36.56^a^	21.28^†^	100.61 ± 55.45^a^	91
KETA	1.95 ± 1.14	0.80	ND	ND	0.59^†^	69
MDMA	4.60 ± 2.90^a^	0.45 ± 0.45^b^	0.60^†^	0.16^†^	1.10 ± 0.45^b^	76*
MA	ND	ND	ND	ND	ND	–
MOR	22.77 ± 17.09^†^	ND	ND	ND	14.10 ± 7.13^†^	–
THC-COOH	ND	ND	ND	ND	ND	–

Dry-season pond-system flow				% red. St. A-C	Temporary pond-system bypass			
	Station A	Station B	Station C		Station A’	Station B’	Station E’	% red. St. A’-E’
n	15	15	10		4	4	4	
AM	3.39 ± 2.37^†^	1.90 ± 2.03^†^	1.42^†^	58	ND	ND	ND	–
BE	704.45 ± 606.95^a^	157.99 ± 110.91^b^	58.49 ± 31.08^c^	91*	385.12 ± 418.91^a^	299.07 ± 340.95^a^	272.71 ± 300.67^a^	24
CAF	5491.56 ± 4234.02^a^	610.00 ± 523.57^b^	326.32 ± 215.70^b^	94*	1787.12 ± 2092.57^a^	728.67 ± 828.63^a^	600.65 ± 575.06^a^	63
COC	93.89 ± 138.86^a^	1.41 ± 0.95^a^	0.56 ± 0.55^a^	99	22.01 ± 28.98^a^	6.92 ± 4.91^b^	4.99 ± 4.28^b^	74*
COT	539.17 ± 335.29^a^	185.20 ± 67.82^a^	116.84 ± 55.00^b^	78*	167.73 ± 202.52^a^	ND	ND	100
KETA	2.58 ± 3.02^a^	0.66 ± 0.45^a^	0.71 ± 0.52^a^	72	0.46 ± 0.23^a^	0.53 ± 0.12^a^	0.43 ± 0.16^a^	0
MDMA	5.66 ± 3.26^a^	1.40 ± 0.60^b^	0.67 ± 0.30^c^	88*	5.57 ± 4.89^a^	4.44 ± 3.92^a^	3.01 ± 2.84^a^	37
MA	0.80 ± 0.75^†^	1.17 ± 1.12^†^	0.51 ± 0.41^†^	35	ND	ND	ND	–
MOR	17.49 ± 19.37^†^	12.48 ± 3.06^†^	ND	100	5.96 ± 5.20^a^	6.26 ± 4.61^a^	5.12 ± 4.27^a^	14
THC-COOH	110.31 ± 69.70^†^	ND	ND	100	ND	ND	ND	–

*AM* Amphetamine; *BE* Benzoylecgonine; *CAF* Caffeine; *COC* Cocaine; *COT* Cotinine; *KETA* Ketamine; *MDMA* Methylenedioxymethamphetamine hydrochloride; *MA* Methamphetamine; *MOR* Morphine; *THC-COOH* 11-nor-Δ^9^-tetrahydrocannabinol-9-carboxylic acid

Significant reductions are indicated by an asterisk (*). *ND *not detected; † statistical comparison not performed (n < 3)

#### Pharmaceuticals and personal care products

Among the analysed PhPCPs analysed, benzophenone 4 (BP4), carbamazepine, and gemfibrozil were the most frequently detected compounds across all sampling stations and hydrological scenarios (Table 6). Acetaminophen and naproxen exhibited the highest mean concentrations, exceeding 2 µg L^−1^ and 1 µg L^−1^, respectively, at the WWTP effluent channel (station A) during all study periods. Gabapentin and sulfamethoxazole were also commonly detected, with mean concentrations above 100 ng L^−1^. In contrast, bezafibrate, fluoxetine, furosemide, and triclosan remained consistently below the method detection limit in all samples. Hydrochlorothiazide was detected in all samples at trace levels (below the limit of quantification, Table S3) and was therefore not considered in further analyses. During the wet-season pond-flow period, BP4, naproxen and trimethoprim exhibited significant reductions between the WWTP effluent channel (station A) and the main wetland basin (station E), with mean removal efficiencies of 77%, 90% and 96%, respectively. Similar spatial reduction patterns were observed during the dry-season pond-flow period. In contrast, PhPCP concentrations remained relatively constant between sampling stations during the temporary bypass period, with no significant spatial gradients detected.

**Table 6:** Mean concentrations (± SD; ng L⁻^1^) of quantified PhPCPs at the sampling stations during the three hydrological scenarios. Different lowercase letters indicate significant differences between sampling stations (*Q*-value < 0.05)

Wet-season pond-system flow						
	Station A	Station B	Station C	Station D	Station E	% red. St. A-E
n	6	6	6	6	6	
ACET	2059.34 ± 3089.93^†^	43.13^†^	765.96^†^	486.37^†^	775.02^†^	63
BP4	1342.01 ± 331.89^a^	560.07 ± 318.00^bc^	656.98 ± 260.12^ab^	265.49 ± 158.72^c^	304.32 ± 208.70^bc^	77*
CBMZ	50.07 ± 16.82^ab^	30.97 ± 16.30^ab^	35.55 ± 9.24^a^	22.64 ± 6.94^b^	41.57 ± 29.44^ab^	16
DICLO	ND	ND	ND	ND	ND	–
GBP	848.87 ± 441.29^a^	396.36 ± 324.98^a^	633.91 ± 596.50^a^	354.65 ± 302.50^a^	267.99 ± 72.93^a^	68
GFB	118.90 ± 75.77^a^	66.51 ± 22.22^a^	65.45 ± 48.97^a^	62.76 ± 29.58^a^	54.89 ± 37.64^a^	53
KETO	42.98 ± 39.13^†^	ND	ND	ND	ND	100
NPX	1646.51 ± 397.80^a^	538.06 ± 392.03^b^	332.97 ± 266.24^b^	143.07 ± 154.24^b^	162.59 ± 97.57^b^	90*
SMX	366.75 ± 178.44^a^	134.48 ± 71.01^a^	169.47 ± 73.28^a^	61.77 ± 31.62^a^	138.11 ± 110.40^a^	62
TRIM	131.74 ± 61.52^ab^	25.69 ± 8.23^a^	10.84 ± 3.25^b^	1.50 ± 1.22^c^	4.93 ± 3.09^c^	96*

Dry-season pond-system flow				% red. St. A-C	Temporary pond-system bypass			
	Station A	Station B	Station C		Station A’	Station B’	Station E’	% red. St. A’-E’
n	15	15	10		4	4	4	
ACET	2571.74 ± 973.53^†^	106.87 ± 148.37^†^	22.56^†^	99	3572.51^†^	41.32^†^	65.89^†^	98
BP4	292.95 ± 249.44^a^	107.23 ± 83.77^b^	148.87 ± 107.44^b^	49*	106.07 ± 32.96^a^	108.31 ± 27.18^a^	108.68 ± 26.39^a^	–
CBMZ	22.28 ± 11.97^a^	22.07 ± 12.39^a^	22.09 ± 9.54^a^	0	16.06 ± 6.68^a^	18.52 ± 2.72^a^	18.47 ± 3.60^a^	–
DICLO	54.57 ± 29.46^a^	14.58 ± 10.28^a^	18.59 ± 11.87^a^	65	73.00 ± 36.36^a^	67.66 ± 28.14^a^	60.88 ± 28.68^a^	16
GBP	409.02 ± 294.88^a^	369.22 ± 418.79^a^	515.58 ± 331.46^a^	–	344.76 ± 374.27^a^	277.54 ± 290.54^a^	227.74 ± 192.85^a^	33
GFB	191.60 ± 81.98^a^	171.53 ± 92.69^a^	198.73 ± 97.63^a^	–	140.38 ± 79.17^a^	163.15 ± 48.78^a^	159.31 ± 47.34^a^	–
KETO	43.74 ± 51.16^†^	ND	ND	100	50.88 ± 60.93^a^	18.75 ± 7.94^a^	11.87 ± 1.62^a^	76
NPX	1036.17 ± 615.35^a^	324.01 ± 242.7^b^	182.51 ± 199.86^c^	82*	1420.44 ± 569.32^a^	1227.90 ± 555.97^a^	946.44 ± 658.69^a^	18
SMX	184.28 ± 168.73^a^	35.42 ± 26.05^a^	39.22 ± 15.98^a^	78	83.13 ± 20.32^a^	94.96 ± 20.55^a^	79.36 ± 21.30^a^	4
TRIM	36.67 ± 23.86^a^	7.80 ± 6.69^b^	5.22 ± 4.96^c^	85*	25.01 ± 18.26^a^	10.82 ± 2.75^a^	9.83 ± 3.34^a^	60

*ACET* Acetaminophen; *BP4* Benzophenone-4; *CBMZ* Carbamazepine; *DICLO* Diclofenac; *GBP* gabapentin; *GFB* Gemfibrozil; *KETO* Ketoprofen; *NPX* Naproxen; *SMX* Sulfamethoxazole; *TRIM* Trimethoprim

Significant reductions are indicated by an asterisk (*). *ND* not detected; † statistical comparison not performed (n < 3)

### Toxicity assessment

**Fig. 3:**
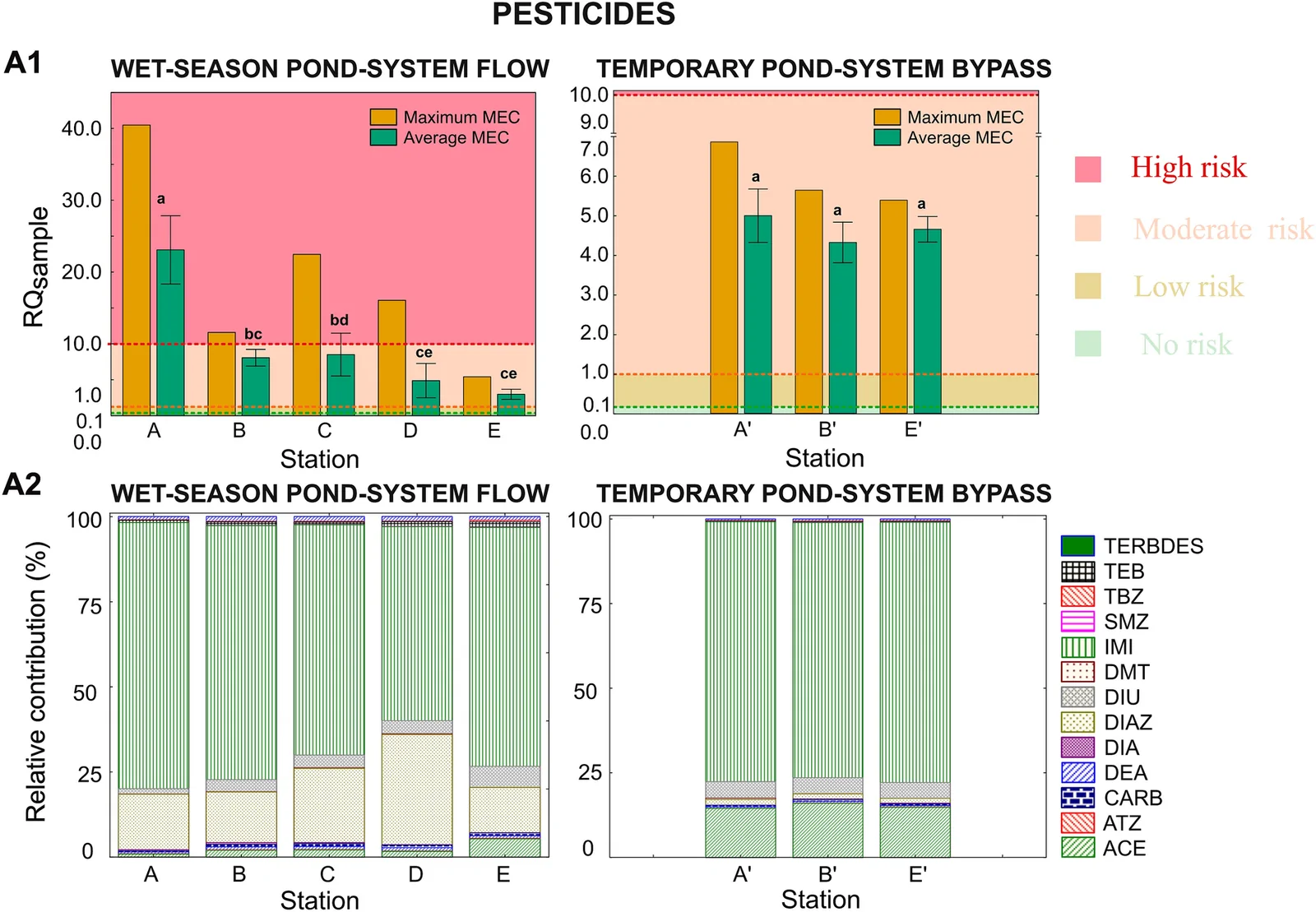
**A1** Cumulative risk quotients (RQ_sample_) calculated from both maximum and average measured environmental concentrations (MECs) of pesticides for each sampling station during the wet-season pond-system flow period (stations A–E) and the temporary pond-system bypass (stations A’–E’). **A2** Relative contribution (%) of each quantified pesticide to the average RQ_sample_ during wet-season pond-system flow and temporary pond-system bypass periods. Compounds with RQ > 10 were classified as high risk, 1 < RQ < 10 as moderate risk, 0.1 < RQ < 1 as low risk, and RQ < 0.1 as no risk

**Fig. 4:**
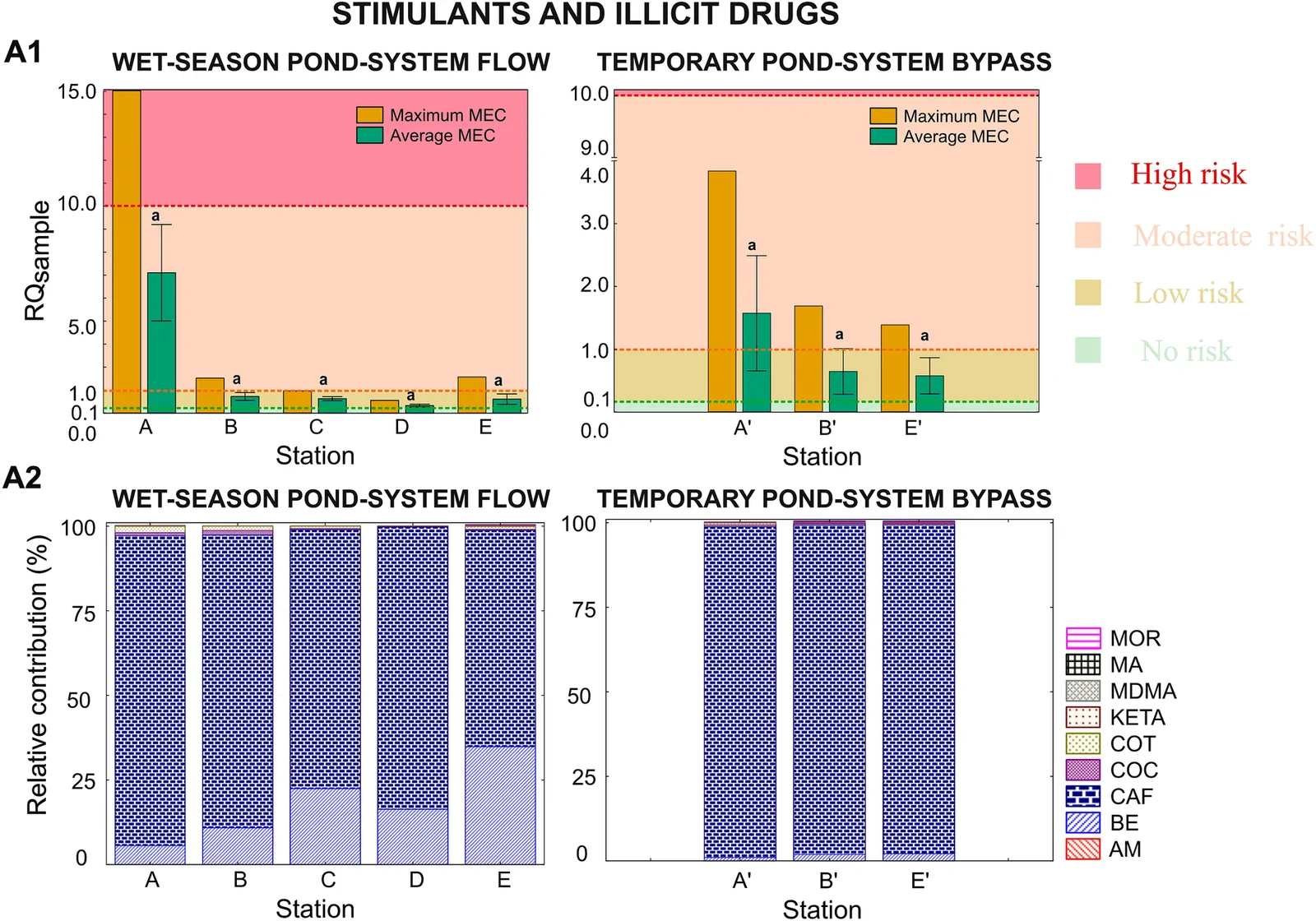
**A1** Cumulative risk quotients (RQ_sample_) calculated from both maximum and average measured environmental concentrations (MECs) of stimulants and illicit drugs for each sampling station during the wet-season pond-system flow period (stations A–E) and the temporary pond-system bypass (stations A’–E’). **A2** Relative contribution (%) of each quantified pesticide to the average RQ_sample_ during wet-season pond-system flow and temporary pond-system bypass periods. Compounds with RQ > 10 were classified as high risk, 1 < RQ < 10 as moderate risk, 0.1 < RQ < 1 as low risk, and RQ < 0.1 as no risk

**Fig. 5:**
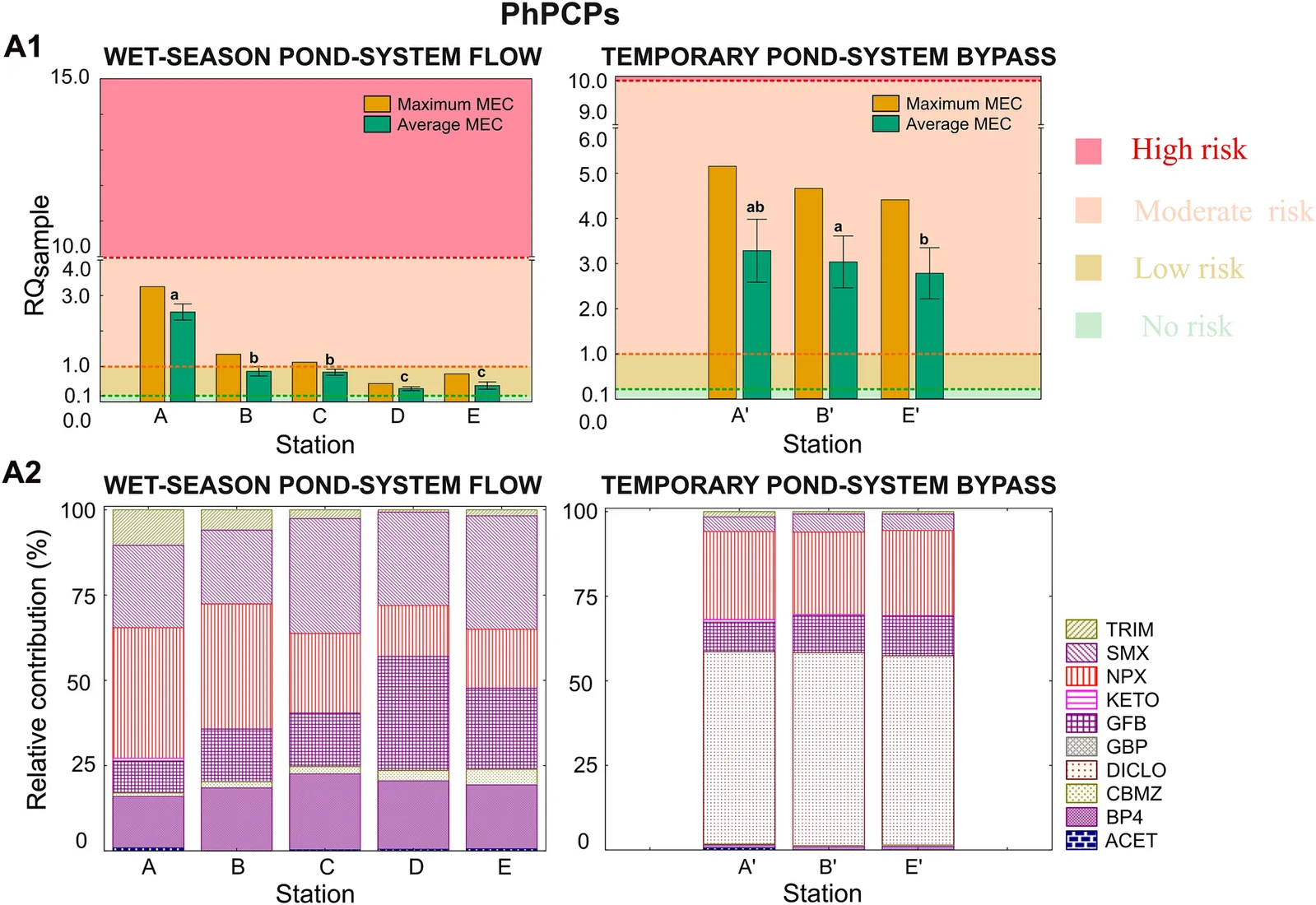
**A1** Cumulative risk quotients (RQ_sample_) calculated from both maximum and average measured environmental concentrations (MECs) of PhPCPs for each sampling station during the wet-season pond-system flow period (stations A–E) and the temporary pond-system bypass (stations A’–E’). **A2** Relative contribution (%) of each quantified pesticide to the average RQ_sample_ during wet-season pond-system flow and temporary pond-system bypass periods. Compounds with RQ > 10 were classified as high risk, 1 < RQ < 10 as moderate risk, 0.1 < RQ < 1 as low risk, and RQ < 0.1 as no risk

RQ_sample_ values were further grouped by compound class: pesticides, stimulants and illicit drugs, and PhPCPs (Fig. 3, 4 and 5, Table S5). During the wet-season pond-flow period, cumulative risk decreased progressively from the WWTP effluent channel (station A) to the Fuente de Piedra main wetland basin (station E). Pesticides represented the dominant risk category, particularly at station A, where RQ_sample_ values exceeded the high-risk threshold (RQ > 10) (Fig. 3A1). Downstream reductions in RQ_sample_ values resulted in moderate overall risk levels at station E (0.1 < RQ < 10). Imidacloprid was the principal contributor to cumulative risk, followed by diazinon and diuron (Fig. 3A2, Table S5). Stimulants and illicit drugs also contributed to ecotoxicological risk at the WWTP effluent channel, primarily through caffeine and benzoylecgonine (Fig. 4). Caffeine reached moderate-risk levels (1 < RQ < 10), whereas benzoylecgonine contributed to a lesser extent. Risk values for this contaminant group declined significantly along the pond network, reaching low-risk levels at station E (*P*-value = 0.043; *Q*-value = 0.109). PhPCPs also contributed to moderate risk at station A, mainly driven by naproxen, gemfibrozil, sulfamethoxazole and BP4 (Fig. 5). Both average and worst-case risks estimates decreased significantly towards the main wetland basin, reaching low-risk levels at station E.

In contrast, during temporary bypass conditions, RQ_sample_ values for pesticides and stimulants and illicit drugs showed no significant spatial variation between the WWTP effluent channel (station A′), the intermediate station (B′), and the main wetland (station E′) (Fig. 3A1 and 4 A1). Risk levels remained within the moderate-risk category throughout the flow pathway. Imidacloprid, acetamiprid, and diuron were the principal contributors to pesticide risk (Fig. 3A2), whereas caffeine and benzoylecgonine dominated the risk associated with stimulants and illicit drugs (Fig. 4A2). PhPCPs exhibited a significant reduction in RQ_sample_ values between stations B’ and E’ (Fig. 5A1); however, overall risk remained withing the moderate-risk category. Diclofenac and naproxen were the primary contributors to PhPCP risk, followed by BP4 (Fig. 5A2).

## Discussion

The results support the initial hypothesis that the semi-natural pond system contributes substantially to the attenuation of nutrient concentrations and wastewater-derived contaminants (pesticides, stimulants and illicit drugs, and PhPCP) in treated wastewater before it reaches Fuente de Piedra main wetland basin. This conclusion is supported by the consistent downstream reductions observed under both pond-system flow conditions investigated in contrast to the constant concentrations observed for most analytes during the temporary pond-system bypass period.

Mediterranean wetlands such as Fuente de Piedra are biodiversity hotspots that are increasingly exposed to anthropogenic pressures, including (un)treated wastewater discharges and agricultural runoff. Although the semi-natural pond system substantially improves water quality, it functions primarily as an attenuation buffer rather than a complete treatment system. Consequently, significant reductions in nutrients and contaminants occur along the pond system, but residual nutrient loads and several wastewater-derived contaminants still reach the main wetland. The mean annual TP concentration at the discharge point (1.32 ± 0.71 mg L^−1^) exceeds by more than an order of magnitude the OECD threshold for hypereutrophic systems (0.10 mg L^−1^) (Vollenweider, 1968). This trophic state, together with the detection of several PhPCP and stimulants (e.g., caffeine, acetaminophen, naproxen, cotinine, benzoylecgonine, BP4, and gabapentin) at concentrations in the hundreds of ng L^−1^, raises concerns regarding potential impacts on aquatic biodiversity. Excessive nutrient loading promotes eutrophication, leading to algal blooms, hypoxia, and shifts in community structure, frequently resulting in the loss of sensitive taxa and reduced diversity (Dodds et al., 2008; Smith et al., 1999). Zooplankton communities, which play a central role in nutrient cycling and food-web dynamics, are particularly sensitive to both nutrient enrichment and chemical stressors (Beklioglu et al., 2003; Jeppesen et al., 2000). In addition, several wastewater-derived pesticides, PhPCP, stimulants and illicit drugs may impair aquatic organisms even at low concentrations by affecting reproduction, development and species interactions (Hejna et al., 2022; Singh et al., 2025). Compounds such as caffeine and naproxen have been shown to alter zooplankton and phytoplankton community structure and functional diversity (Chia et al., 2021; Rodrigues et al., 2025; Rosi-Marshall et al., 2013; Zhao et al., 2024). Therefore, although the semi-natural pond system substantially attenuates nutrient and contaminant loads, the concentrations reaching the wetland remain sufficiently elevated to warrant ecological concern. Given the continuous discharge of nutrient-rich and contaminant-loaded effluents, the combined effects of hypereutrophic conditions and chemical stressors may contribute to ecological stress and potentially contribute to biodiversity loss in Fuente de Piedra. These impacts may be further amplified by the Mediterranean hydrological regime, characterized by strong fluctuations in water level and salinity, which can increase the vulnerability of aquatic communities (García & Niell, 1993; García et al., 1997).

Although the Fuente de Piedra WWTP mainly treats urban wastewater, the occurrence of pesticides in treated effluents is not unexpected. Previous studies have documented the presence of pesticides in municipal wastewater due to a combination of domestic uses (e.g., insecticides and biocides), urban runoff, and diffuse inputs from surrounding agricultural areas reaching sewer networks and receiving waters (Köck-Schulmeyer et al., 2013; Wittmer et al., 2010). The detection of neonicotinoids and triazine-related compounds in the present study is therefore consistent with contaminant profiles commonly reported in urban-treated wastewater effluents (Sadaria et al., 2016). Given the predominantly agricultural landscape surrounding Fuente de Piedra, and the WWTP design pond-based treatment diffuse agricultural inputs into the wastewater treatment system and downstream aquatic environments cannot be discarded (Masiá et al., 2013). In agreement with this interpretation, acetamiprid and imidacloprid were the most abundant pesticides detected, while triazines and their degradation products were consistently found throughout the pond system. Several of these compounds exhibited substantial attenuation during pond-system flow conditions, whereas little or no attenuation was observed during the temporary pond-system bypass period, highlighting the importance of hydraulic connectivity and residence time for pesticide attenuation.

Most studies assessing the performance of constructed wetlands have primarily focused on nutrient removal (e.g., Rodrigo et al., 2013; de-los-Ríos-Mérida et al., 2017, 2021; Hernández-Crespo et al., 2017), whereas comparatively few have examined EC attenuation (Gorito et al., 2017; Overton et al., 2023; Wolff et al., 2024). The efficiency of EC removal in wetland systems is influenced by multiple interacting biotic (e.g., microbial communities, vegetation) and abiotic factors (e.g., temperature, pH, redox conditions and hydrological regime) (Muduli et al., 2023). This complexity makes it challenging to fully understand how water circulation specifically impacts the overall improvement in water quality. Although the present study focused on evaluating spatial changes under contrasting hydrological configurations, complementary multivariate analyses integrating physicochemical variables, nutrients, and EC concentrations could provide additional insight into the environmental gradients and mechanisms controlling contaminant attenuation in Mediterranean semi-natural pond systems. Among these factors, hydrological variables are particularly critical. Hydraulic loading rate (HLR) and hydraulic retention time (HRT) largely determine system performance. Elevated HLR promotes rapid wastewater transport through the wetland, reducing HRT and limiting both oxygen transfer and contact time for aerobic degradation and sorption processes (Ilyas & van Hullebusch, 2019). In Fuente de Piedra, spatial changes in water chemistry were strongly associated with circulation time between upstream and downstream stations. During the wet-season pond-system flow period, HRT in Laguneto ranged from 26 to 49 days, reaching 61 days in February 2023. That month was characterized by minimal rainfall (0.4 mm d⁻^1^), reduced WWTP inflow (534.9 m^3^ d⁻^1^), and high evaporation rates (2.23 L m⁻^2^ d⁻^1^), conditions that explain the prolonged retention time. During dry-season pond-system flow period, HRT varied between 40 and 54 days. Under these scenarios, extended HRT facilitated substantial reductions in nutrients and ECs concentrations, although complete removal was not achieved. In contrast, during temporary pond-system bypass, HTT decreased to approximately 16 h, and no significant spatial changes in nutrient or EC concentrations were observed between the most distant stations. Similarly, de-los-Ríos-Mérida et al. (2021) reported limited nutrient removal (7% for TP and 35% for TN) in treated wastewater discharged through the Charcón creek at the same study site (Fuente de Piedra), attributing the low efficiency to the short HRT. A comparison with previous data from Fuente de Piedra (de-los-Ríos-Mérida et al., 2017) suggest changes in long-term system performance. Although direct comparisons are constrained by differences in sampling design, TN removal efficiency increased substantially (from 50 to 75%), whereas TP removal declined (from 40 to 21%). These contrasting trends highlight the dynamic nature of nutrient processing in semi-natural wetland systems and the importance of hydrological management.

Water circulation within the semi-natural pond system is strongly modulated by seasonal variability, a feature that is particularly pronounced under Mediterranean climatic conditions. Although treated wastewater is continuously discharged from the WWTP throughout the year, hydraulic connectivity within the pond system progressively decreases during late spring and summer. As water levels decline, the downstream ponds (Laguna de los Juncares and Los Juncares) gradually dry out, preventing the treated effluent from reaching Fuente de Piedra main wetland basin during the driest periods. During the wet season, TN and DIP concentrations decreased significantly from the WWTP discharge point (station A) to the Laguna de los Juncares downstream section (station C), with reductions of 60% and 65%, respectively. In the dry season, TN also declined significantly (44%), whereas larger reductions were observed for TP (46%) and DIP (79%). These seasonal contrasts reflect differences in hydrological and biogeochemical functioning. These patterns likely reflect the combined influence of hydrological conditions and seasonally varying environmental factors, including solar radiation, evaporation rates, vegetation development, and microbial activity, all of which may affect nutrient cycling and contaminant attenuation. In addition to hydrological conditions, spatial and seasonal changes in dissolved oxygen, conductivity, nutrient concentrations, and ionic composition may also have influenced contaminant attenuation by modifying microbial activity, chemical speciation, and sorption processes. Although the study period was characterized by relatively dry climatic conditions compared with the long-term regional average, the distinction between the wet-season and dry-season pond-system flow scenarios remained consistent with the observed hydraulic connectivity and wastewater circulation patterns within the semi-natural pond system. Therefore, the differences observed between hydrological scenarios reflect changes in the actual functioning of the system rather than being solely attributable to interannual climatic variability. Lower flow rates and extended HRT during the dry season enhance nutrient removal through sedimentation, microbial uptake, and denitrification (Kadlec & Wallace, 2009; Mitsch & Gossilink, 2000). Higher temperatures further stimulate nutrient assimilation and microbial activity associated with periphyton, biofilms, and emergent vegetation (Reddy & D’Angelo, 1997). As previously described, the semi-natural pond system supports extensive helophytic vegetation that likely contributes to nutrient and contaminant attenuation through multiple pathways, including sediment trapping, nutrient uptake, provision of surfaces for biofilm development, and enhancement of microbial transformation processes. Although biochemical oxygen demand (BOD) was not measured and therefore seasonal changes in organic matter production and decomposition could not be directly assessed, TSS were monitored throughout the study and did not differ significantly among sampling stations under any water-circulation scenario. This suggests that the observed reductions in nutrients and contaminants cannot be explained solely by particulate matter removal. Instead, a combination of biological uptake, microbial processing, sediment–water interactions, and extended hydraulic residence times likely contributed to the attenuation observed under pond-system flow conditions. Conversely, during the wet season, higher flow velocities and shorter residence times limit contact between water column and reactive sediments or vegetation, reducing opportunities for nutrient transformation and retention (Kovacic et al., 2000). A comparable seasonal pattern was observed for ECs. During the wet season, only a limited number of compounds (desethylatrazine, terbuthylazine-desethyl, naproxen, and trimethoprim) decreased significantly between stations A and C, whereas stimulants or illicit drugs showed no significant reduction. In contrast, during the dry season, a broader range of ECs exhibited significant reductions along the same transect, including several pesticides (atrazine, carbendazim, diuron, diazinon, simazine, tebuconazole, terbuthylazine, and terbuthylazine-desethyl), stimulants and illicit drugs (benzoylecgonine, caffeine, cotinine, and MDMA), and PhPCPs (BP4, naproxen, and trimethoprim). This enhanced removal during the dry season is consistent with longer HRT and greater opportunities for photodegradation, microbial transformation, and sorption at the sediment–water interface (Matamoros et al., 2009; Vieno et al., 2007).

A well-documented Mediterranean example is Tancat de la Pipa (Albufera de Valencia, Spain), a 40-ha free-water-surface constructed wetland established in 2009 on former rice fields to comply with the European Union Water Framework Directive and restore degraded habitats. Its dual objectives were to improve the quality of the hypereutrophic waters of the Albufera de Valencia lagoon, the largest coastal lagoon in the Iberian Peninsula, and to enhance biodiversity. Extensive research has evaluated water quality, plankton communities, and vegetation dynamics at this site (e.g., Calero et al., 2015; Hernández-Crespo et al., 2017; Rodrigo & Carabal, 2020; Rodrigo & Segura, 2020; Rodrigo et al., 2013, 2015, 2018). Rodrigo et al. (2013) reported retention efficiencies of 42% for TSS, 30% for TN, and 49% for TP, indicating slightly higher TP but considerably lower TN removal than in Fuente de Piedra, underscoring site-specific differences in nutrient retention. Comparisons with other Mediterranean wetlands reveal similar patterns in ECs behaviour. Martínez-Megías et al. (2024) reported highly variable EC removal in a free-water-surface constructed wetland in southeastern Spain, with carbamazepine and diclofenac showing limited attenuation (< 20%), whereas caffeine and ketoprofen were efficiently removed (> 80%). These findings are consistent with our results, where carbamazepine exhibited negligible reduction, while caffeine and ketoprofen were reduced by 93–100% under both wet and dry circulation scenarios. The concordance across systems suggests that EC removal efficiency is largely governed by compound-specific physicochemical properties, such us persistence, sorption affinity and susceptibility to biodegradation, although hydrological regime remains a key modulating factor.

Removal of ECs in Fuente de Piedra semi-natural pond system reflects the combined action of degradative processes (primarily photolysis and microbial biodegradation) and, to a lesser extent, sorption to sediments. As reported for other wetland systems (Carvalho et al., 2014; Matamoros et al., 2009; Zhang et al., 2015), attenuation efficiency depends not only on wetland configuration but also compound-specific properties. Compounds with shorted half-lives (DT50), generally exhibited higher removal efficiencies, whereas more persistent substances showed limited attenuation. Mass-balance calculations between stations A (WWTP outlet) and B (Laguneto outlet) (Matamoros et al., 2015; see Text S1), indicated that stimulants and illicit drugs experienced greater attenuation under both pond-system conditions compared with PhPCPs and pesticides (Fig. S2). The negative relationship observed between DT50 values (Table S6) and removal efficiencies under usual and modified circulation scenarios (Fig. S3) supports this pattern. For example, cotinine, caffeine, and cocaine, which are characterized by relatively short half-lives, consistently showed high removal under both pond-system conditions. Similar responses were observed for ketoprofen, trimethoprim, naproxen, imidacloprid and diazinon. In contrast, more recalcitrant compounds such as carbamazepine exhibited negligible removal. Sorption processes may additionally contribute to the attenuation of moderately to highly hydrophobic compounds. For instance, gemfibrozil (logKow > 4) may partly accumulate in sediments (Fig. S5). ECs with intermediate hydrophobicity (2 ≤ logKow ≤ 4), including atrazine, tebuconazole, naproxen and ketoprofen, likely undergo a combination of biodegradation, photolysis and partial sorption, resulting in variable removal efficiencies across scenarios. Highly hydrophilic compounds (log Kow < 2) are more dependent on degradative pathways; caffeine (logKow = − 0.07) exemplifies this behaviour, showing consistently high attenuation under usual circulation conditions. Although sorption reduces aqueous concentrations, sediment-associated ECs may persist and potentially remobilize during extreme hydraulic events, representing a delayed ecological risk. During the temporary bypass period, when water bypassed the ponds and flowed directly to Fuente de Piedra main wetland basin, the relationship between half-lives or sorption capacity and removal efficiency were weak and non-significant (Spearman’s ρ = − 0.389, *P*-value > 0.05 for DT50; Pearson’s *r* = − 0.29; *P*-value > 0.05 for sorption capacity). This indicates that reduced residence time limits the operation of biogeochemical attenuation processes (Ávila et al., 2010; Gros et al., 2017). Overall, these findings highlight the importance of sufficient hydraulic retention and active microbial communities for effective ECs mitigation, particularly for compounds with shorter persistence (Hijosa-Valsero et al., 2011; Verlicchi & Zambello, 2014).

Although the semi-natural pond system substantially improves water quality, nutrient and EC concentrations remain elevated at the discharge point into the Fuente de Piedra main wetland basin. The ecotoxicological patterns derived from RQ_sample_ values provide further insight into system performance under contrasting hydrological regimes. During wet-season pond-flow period, the progressive decline in RQ_sample_ values from station A to station E demonstrates the capacity of the semi-natural ponds to attenuate contaminant loads, particularly pesticides. This reduction likely reflects the combined influence of dilution, microbial degradation and sedimentation processes operating under adequate HRT (e.g., Luo et al., 2014; Grenni et al., 2018). The dominant contribution of imidacloprid and diazinon to overall risk is consistent with their persistence and high toxicity to aquatic invertebrates (Jemec et al., 2007). In contrast, the limited spatial change in RQ_sample_ values during temporary bypass indicates that bypassing the semi-natural pond system via direct flow to Fuente de Piedra main wetland basin compromises its attenuation capacity. Under these modified conditions, rapid transport reduces opportunities for degradation, and moderate risk levels persist across stations. This pattern underscores the importance of maintaining hydrological connectivity to sustain ecological filtering functions. Importantly, reductions in concentration did not always translate into low-risk conditions. Compounds such as acetamiprid, carbendazim and gemfibrozil maintained moderate or high RQ_sample_ values due to their low PNECs. This highlights the need for compound-specific risk assessments rather than relying solely on concentration-based metrics. Finally, the RQ_sample_ approach has inherent limitations. It does not account for differences in modes of action, potential mixture interactions (synergistic and antagonistic effects), or differences in species sensitivity. Furthermore, it excludes non-target ECs and is based on grab samples that provide only a snapshot of exposure conditions. Because ecotoxicological thresholds specific to saline and hypersaline environments are currently unavailable for most compounds, the PNEC values used in this assessment correspond to freshwater organisms, whose sensitivity may differ from that of the biota inhabiting the study area (Gaw et al., 2014; Leung et al., 2001). This source of uncertainty is particularly relevant for the Fuente de Piedra main wetland basin, where salinity reaches hypersaline conditions, whereas the upstream semi-natural pond system is characterized by substantially lower salinity. Accordingly, the risk estimates presented in this study should be interpreted with caution, as freshwater-derived PNECs may not fully reflect the ecological sensitivity of the biological communities inhabiting the hypersaline Fuente de Piedra main wetland basin (Hall & Anderson, 1995; Szöcs et al., 2012).

In addition to point-source inputs, Fuente de Piedra wetland receives nutrients and contaminants from groundwater (Montalván et al., 2017), and through guanotrophication. Waterbirds, particularly gulls, import nutrients (N and P) from surrounding areas such as landfills (Batanero et al., 2017; Martín-Vélez et al., 2019). Using GPS tracking, Martín-Vélez et al. (2019) estimated annual inputs of 10.17 kg N ha⁻^1^ yr⁻^1^ and 2.07 kg P ha⁻^1^ yr⁻^1^, with regurgitated pellets representing a greater phosphorus source than faeces. These inputs have reportedly increased in recent years. The potential role of waterbirds in mediating nutrient and contaminant dynamics within the treatment pond system upstream of Fuente de Piedra remains largely unexplored. Seasonal censuses of waterbird abundance and habitat use, coupled with nutrient and contaminant monitoring, would help clarify the contribution of waterbirds to nutrient loading and overall wetland biogeochemistry.

Although the present findings demonstrate the substantial capacity of semi-natural pond systems to improve water quality, the persistence of elevated nutrient concentrations and several wastewater-derived contaminants at the wetland inlet indicates that additional management measures may be required to further reduce contaminant loads reaching protected aquatic systems. Potential approaches may include improvements in wastewater treatment performance, optimization of hydraulic management within treatment systems, source-control strategies, or the integration of complementary remediation technologies. Previous studies have shown that advanced nutrient and contaminant mitigation approaches can enhance water-quality improvement and contribute to resource recovery and circular-economy strategies (Álvarez-Manzaneda et al., 2021a, 2021b; de Vicente et al., 2010; Funes et al., 2016, 2018). Further research is needed to evaluate the feasibility, long-term effectiveness, and ecological implications of such measures under Mediterranean conditions. The relevance of such integrated approaches is likely to increase under future climate scenarios, as warming and altered hydrological regimes are expected to intensify eutrophication processes (Jeppesen et al., 2010), especially in vulnerable Mediterranean wetlands (de Vicente, 2021).

## Conclusions

This study shows that the semi-natural pond system upstream of Fuente de Piedra main wetland basin acts an important attenuation buffer, reducing nutrient concentrations and the occurrence of a wide range of pesticides, stimulants and illicit drugs, and PhPCPs before water reaches the main wetland basin. Attenuation was strongly associated with water circulation and hydraulic residence time, with substantial reductions observed under regular wet- and dry-season pond-system flow conditions but minimal attenuation during the temporary pond-system bypass configuration. Despite these reductions, phosphorus concentrations at the Fuente de Piedra main wetland basin inlet remained above hypereutrophic thresholds, and several persistent ECs (e.g., carbamazepine, gemfibrozil, and acetamiprid) remained associated with moderate to high ecological risks, highlighting that attenuation within the pond system does not fully eliminate wastewater-derived pressures. The observed patterns likely reflect the combined influence of hydrological conditions and other seasonally varying environmental factors. Overall, these findings highlight the ecological and functional importance of maintaining adequate residence times within semi-natural pond systems and support the implementation of complementary management actions aimed at reducing nutrient and contaminant loads reaching protected Mediterranean wetlands. Such measures may enhance ecosystem resilience and help safeguard biodiversity under increasing anthropogenic and climatic pressures.

## Supplementary Information

Below is the link to the electronic supplementary material.

Supplementary file1 (DOCX 1457 KB)

## Data Availability

The datasets generated and analysed during the current study are not publicly available but are available from the corresponding author upon reasonable request.
