# Correction: Nature-based solutions for nutrient and emerging contaminant reduction in a Mediterranean Ramsar wetland

**DOI:** 10.1007/s10653-026-03493-1

**Published:** 2026-09-29

**Authors:** Emilio Moreno, María Gómez-Pozuelos, Francisco Guerrero, Cristina Postigo, África Lupión, José María Conde-Porcuna, Inmaculada de Vicente

**Affiliations:** 1 Institute of Water Research, University of Granada, Ramón y Cajal, 4, 18071 Granada, Spain; 2 Department of Animal Biology, Plant Biology and Ecology, University of Jaén, Campus Las Lagunillas s/n, 23071 Jaén, Spain; 3 Center for Advanced Studies in Earth Sciences, Energy and Environment, University of Jaén, Campus Las Lagunillas s/n, 23071 Jaén, Spain; 4 Department of Civil Engineering, School of Civil Engineering, University of Granada, Avenida de Fuentenueva s/n, 18071 Granada, Spain; 5 Consejería de Sostenibilidad, Medio Ambiente y Economía Azul (Regional Ministry of Sustainability, Environment and Blue Economy), Reserva Natural Laguna Fuente de Piedra (Fuente de Piedra Nature Reserve), 29520 Fuente de Piedra, Spain; 6 Department of Ecology, University of Granada, Avenida de Fuentenueva s/n, 18071 Granada, Spain

**Correction to: Environ Geochem Health (2026) 48:511** https://doi.org/10.1007/s10653-026-03377-4

In this article, Dr. Francisco Guerrero was inadvertently omitted from the list of contributors responsible for investigation, data collection, and formal analysis. The 'Author Contributions' statement has been revised to read as follows:

All authors contributed to the study conception and design. Investigation, data collection, and formal analysis were performed by E. Moreno, M. Gómez-Pozuelos, F. Guerrero, J. M. Conde, and I. de Vicente. Analysis of emerging contaminants was performed by C. Postigo. A. Lupión facilitated fieldwork within the Fuente de Piedra Nature Reserve. The first draft of the manuscript was written by Emilio Moreno and Inmaculada de Vicente, and all authors commented on previous versions of the manuscript. All authors read and approved the final manuscript.

The original article has been corrected.

