# Supplementary material for: Nature-based solutions for nutrient and emerging contaminant reduction in a Mediterranean Ramsar wetland
Source: Environ Geochem Health. 2026 Aug 8;48(12):511. doi: 10.1007/s10653-026-03377-4 (PMC13452764; doi:10.1007/s10653-026-03377-4)
Supplement: Supplementary file 1 [file 10653_2026_3377_MOESM1_ESM.docx]

**Supplementary information**

**Nature-based solutions for nutrient and emerging contaminant reduction in a Mediterranean Ramsar wetland**

**Emilio Moreno^a*^, María Gómez-Pozuelos^a^, Francisco Guerrero^b,c^, Cristina Postigo^a,d^, África Lupión^e^, José María Conde-Porcuna^a,f^ and Inmaculada de Vicente^a,f^**

^a^ Institute of Water Research, University of Granada, Ramón y Cajal, 4, 18071 Granada, Spain

^b^ Department of Animal Biology, Plant Biology and Ecology, University of Jaén, Campus Las Lagunillas s/n, 23071 Jaén, Spain

^c^ Center for Advanced Studies in Earth Sciences, Energy and Environment, University of Jaén, Campus Las Lagunillas s/n, 23071 Jaén, Spain

^d^ Department of Civil Engineering, School of Civil Engineering, University of Granada, Avenida de Fuentenueva s/n, 18071 Granada, Spain

^e^ Consejería de Sostenibilidad, Medio Ambiente y Economía Azul (Regional Ministry of Sustainability, Environment and Blue Economy), Reserva Natural Laguna Fuente de Piedra (Fuente de Piedra Nature Reserve), 29520 Fuente de Piedra, Spain

^f^ Department of Ecology, University of Granada, Avenida de Fuentenueva s/n, 18071 Granada, Spain

* Corresponding author: [emiliojml@ugr.es](mailto:emiliojml@ugr.es) (ORCID: 0000-0001-6027-7333)

**Table S1** – LC-MS/MS analysis of the target analytes and isotopically labelled compounds determined in the positive ionization mode.

| Analyte | Retention time (min) | Parent  ion (m/z) | Product  ion (m/z) | Fragmentor (V) | Collision energy (V) | SRM1/  SRM2 |
| --- | --- | --- | --- | --- | --- | --- |
| 6-monoacetylmorphine | 5.76 | 328 | 165 | 160 | 46 | 62 |
|  |  | 328 | 152 | 160 | 86 |  |
| *6-monoacetylmorphine-d3* | *5.76* | *331* | *152* | *136* | *86* |  |
| 4-androstenedione | 11.91 | 287 | 97 | 112 | 26 | 59 |
|  |  | 287 | 109 | 112 | 22 |  |
| Acetaminophen | 5.81 | 152 | 110 | 88 | 18 | 58 |
|  |  | 152 | 65 | 88 | 34 |  |
| *Acetaminophen-d4* | *5.80* | *156* | *114* | *88* | *18* |  |
| Acetamiprid | 7.36 | 223 | 126 | 88 | 26 | 47 |
|  |  | 223 | 56 | 88 | 14 |  |
| Amphetamine | 5.68 | 136 | 91 | 64 | 18 | 40 |
|  |  | 136 | 119 | 64 | 10 |  |
| *Amphetamine-d11* | *5.65* | *147* | *98* | *64* | *18* |  |
| Atrazine | 10.58 | 216 | 174 | 112 | 18 | 41 |
|  |  | 216 | 68 | 112 | 46 |  |
| *Atrazine-d5* | *10.53* | *221* | *179* | *112* | *18* |  |
| Benzoylecgonine | 6.59 | 290 | 168 | 88 | 18 | 56 |
|  |  | 290 | 77 | 112 | 66 |  |
| *Benzoylecgonine-d3* | *6.57* | *298* | *171* | *112* | *22* |  |
| Caffeine | 6.54 | 195 | 138 | 112 | 22 | 15 |
|  |  | 195 | 83 | 112 | 34 |  |
| *Caffeine-d9* | *6.50* | *204* | *144* | *88* | *22* |  |
| Carbamazepine | 9.83 | 237 | 194 | 112 | 22 | 17 |
|  |  | 237 | 179 | 112 | 42 |  |
| Carbendazim | 5.91 | 192 | 160 | 88 | 22 | 22 |
|  |  | 192 | 132 | 88 | 34 |  |
| *Carbendzim-d3* | *5.89* | *195* | *160* | *88* | *22* |  |
| Clothianidin | 7.08 | 250 | 169 | 64 | 10 | 56 |
|  |  | 250 | 132 | 64 | 18 |  |
| *Clothianidin-d3* | *7.07* | *253* | *172* | *64* | *10* |  |
| Cocaine | 6.29 | 304 | 182 | 112 | 18 | 35 |
|  |  | 304 | 77 | 112 | 70 |  |
| *Cocaine-d3* | *6.29* | *307* | *185* | *112* | *22* |  |
| Cotinine | 1.67 | 177 | 80 | 88 | 26 | 24 |
|  |  | 177 | 98 | 88 | 26 |  |
| *Cotinine-d3* | *1.65* | *180* | *80* | *112* | *34* |  |
| Desethyl-atrazine | 7.74 | 188 | 146 | 88 | 18 | 26 |
|  |  | 188 | 79 | 88 | 26 |  |
| *Desethyl-atrazine-d6* | *7.69* | *194* | *147* | *88* | *18* |  |
| Desisopropyl-atrazine | 6.81 | 174 | 68 | 160 | 34 | 59 |
|  |  | 174 | 96 | 160 | 18 |  |
| *Desisopropyl-atrazine-d5* | *6.77* | *179* | *69* | *112* | *38* |  |
| Diazinon | 14.2 | 305 | 169 | 112 | 22 | 58 |
|  |  | 305 | 153 | 112 | 22 |  |
| *Diazinon-d10* | *14.1* | *315* | *170* | *112* | *22* |  |
| Dichlorvos | 8.9 | 221 | 79 | 160 | 49 | 65 |
|  |  | 223 | 79 | 160 | 49 |  |
| Dimethoate | 7.3 | 230 | 125 | 64 | 22 | 100 |
|  |  | 230 | 199 | 64 | 10 |  |

**Table S1** (continued)

| Analyte | Retention time (min) | Parent  ion (m/z) | Product  ion (m/z) | Fragmentor (V) | Collision energy (V) | SRM1/  SRM2 |
| --- | --- | --- | --- | --- | --- | --- |
| Diuron | 11.13 | 235 | 72 | 88 | 26 | 155 |
|  |  | 233 | 72 | 88 | 22 |  |
| *Diuron-d6* | *11.10* | *239* | *78* | *88* | *30* |  |
| Fluometuron | 10.26 | 233 | 145 | 64 | 42 | 93 |
|  |  | 233 | 160 | 64 | 30 |  |
| Fluoxetine | 9.39 | 310 | 44 | 64 | 10 | 4 |
|  |  | 310 | 148 | 64 | 10 |  |
| *Fluoxetine-d6* | *9.35* | *316* | *154* | *64* | *10* |  |
| Gabapentin | 5.69 | 172 | 154 | 88 | 14 | 54 |
|  |  | 172 | 137 | 88 | 18 |  |
| Imidacloprid | 6.95 | 256 | 209 | 64 | 18 | 115 |
|  |  | 256 | 175 | 64 | 18 |  |
| Ketamine | 6.10 | 238 | 125 | 88 | 34 | 34 |
|  |  | 238 | 207 | 88 | 14 |  |
| *Ketamine-d4* | *6.10* | *242* | *129* | *88* | *34* |  |
| Ketoprofen | 11.58 | 255 | 209 | 120 | 14 | 73 |
|  |  | 255 | 105 | 120 | 26 |  |
| Malathion | 12.54 | 331 | 127 | 64 | 10 | 70 |
|  |  | 331 | 99 | 64 | 22 |  |
| MDMA | 5.86 | 194 | 163 | 64 | 10 | 41 |
|  |  | 194 | 105 | 64 | 26 |  |
| *MDMA-d5* | *5.86* | *199* | *165* | *64* | *10* |  |
| Methamphetamine | 5.78 | 150 | 91 | 64 | 18 | 40 |
|  |  | 150 | 119 | 64 | 10 |  |
| *Methamphetamine-d14* | *5.74* | *164* | *98* | *64* | *26* |  |
| Morphine | 2.75 | 286 | 152 | 160 | 70 | 54 |
|  |  | 286 | 77 | 160 | 110 |  |
| *Morphine-d3* | *2.71* | *289* | *165* | *160* | *42* |  |
| Parathion methyl | 12.03 | 264 | 232 | 88 | 18 | 95 |
|  |  | 264 | 125 | 88 | 18 |  |
| Simazine | 9.29 | 202 | 124 | 112 | 18 | 11 |
|  |  | 202 | 68 | 112 | 38 |  |
| Sulfamethoxazole | 6.85 | 254 | 92 | 88 | 30 | 103 |
|  |  | 254 | 65 | 88 | 58 |  |
| *Sulfamethoxazole-d4* | *6.841* | *258* | *96* | *88* | *34* |  |
| Tebuconazole | 14.07 | 308 | 70 | 112 | 26 | 3 |
|  |  | 308 | 151 | 112 | 26 |  |
| *Tebuconazole-d6* | *14.04* | *314* | *72* | *112* | *26* |  |
| Terbuthylazine-desethyl | 9.69 | 202 | 146 | 88 | 18 | 0.1 |
|  |  | 204 | 146 | 88 | 14 |  |
| *Terbuthylazine-desethyl-d9* | *9.61* | *211* | *147* | *88* | *18* |  |
| Terbuthylazine | 12.16 | 230 | 174 | 88 | 18 | 23 |
|  |  | 230 | 68 | 88 | 46 |  |
| Testosterone | 12.63 | 289 | 97 | 112 | 26 | 81 |
|  |  | 289 | 109 | 112 | 26 |  |
| *Testosterone-d3* | *12.61* | *292* | *109* | *112* | *26* |  |
| Theophylline | 6.23 | 181 | 124 | 112 | 18 | 31 |
|  |  | 181 | 69 | 112 | 34 |  |
| Thiacloprid | 7.91 | 253 | 126 | 88 | 26 | 26 |
|  |  | 253 | 90 | 88 | 46 |  |
| Thiamethoxam | 6.46 | 292 | 211 | 64 | 10 | 48 |
|  |  | 292 | 181 | 64 | 22 |  |
| Trimetoprim | 5.89 | 291 | 275 | 136 | 26 | 171 |
|  |  | 291 | 123 | 136 | 30 |  |
| *Trimetoprim-d3* | *5.88* | *294* | *123* | *136* | *26* |  |

**Table S2 –** LC-MS/MS analysis of the target analytes and isotopically labelled compounds determined in the negative ionization mode.

| **Analyte** | **Retention time (min)** | **Parent**  **ion (m/z)** | **Product**  **ion (m/z)** | **Fragmentor (V)** | **Collision energy (V)** | **SRM1/**  **SRM2** |
| --- | --- | --- | --- | --- | --- | --- |
| Bezofibrate | 10.17 | 360 | 88 | 274 | 18 | 28 |
|  |  | 360 | 88 | 154 | 34 |  |
| *Bezofibrate-d6* | *10.14* | *366* | *88* | *274* | *18* |  |
| Benzophenona-4 | 8.02 | 307 | 136 | 291 | 30 | 184 |
|  |  | 307 | 136 | 211 | 42 |  |
| Diclofenac | 11.23 | 296 | 64 | 252 | 22 | 12 |
|  |  | 294 | 64 | 250 | 10 |  |
| *Diclofenac-d6* | *11.27* | *300* | *64* | *256* | *10* |  |
| Furosemide | 8.3 | 329 | 88 | 285 | 14 | 55 |
|  |  | 329 | 88 | 205 | 22 |  |
| Gemfibrozil | 13.34 | 249 | 88 | 127 | 10 | 7 |
|  |  | 249 | 88 | 121 | 14 |  |
| Hydrochlorothiazide | 2.13 | 296 | 136 | 269 | 22 | 62 |
|  |  | 296 | 136 | 205 | 22 |  |
| Naproxen | 10.14 | 229 | 40 | 170 | 14 |  |
|  |  | 229 | 40 | 169 | 34 |  |
| *Naproxen-13C-d3* | *10.11* | *233* | *64* | *169* | *38* | 96 |
| (±)-11-nor-9-Carboxy-Δ9-THC | 13.66 | 343 | 136 | 299 | 22 | 27 |
|  |  | 343 | 136 | 245 | 34 |  |
| *(±)-11-nor-9-Carboxy-Δ9-THC -d9* | *13.63* | *352* | *136* | *308* | *22* |  |
| Triclosan | 13.52 | 289 | 64 | 37 | 22 | 33 |
|  |  | 287 | 64 | 35 | 56 |  |

**Table S3** – Method performance in terms of the SPE and LC-MS/MS method in terms of linearity, accuracy, precision, matrix effects, and sensitivity in surface water. (See details in the table footnote)

|  | **Linearity** | | **Accuracy and precision*** | | | | | | | | | | **Sensitivity** | | **Matrix effects** | | **IS used** |
| --- | --- | --- | --- | --- | --- | --- | --- | --- | --- | --- | --- | --- | --- | --- | --- | --- | --- |
|  | R2 | Range  (ng/mL) | 100 mL extraction – 400 ng/L analytes and 100 ng/L IS | | | | 500 mL extraction  – 700 ng/L analytes + 80 ng/L IS | | | | 500 mL extraction –  7 ng/L analytes + 80 ng/L IS | | 100 mL | 500 mL | 100 mL | 500 mL |  |
|  |  |  | Recovery (%) | RSD  (%) | Trueness  (%) | RSD  (%) | Recovery (%) | RSD  (%) | Trueness  (%) | RSD  (%) | Trueness  (%) | RSD  (%) | LOQ  (ng/L) | LOQ  (ng/L) | % | % |  |
| 6-monoacetyl-morphine | 0.9951 | 0.05-500 | 99.9 | 1.2 | 116 | 6.9 | 102.6 | 10.7 | 95.52 | 3.7 | 104.8 | 5.8 | 2.5 | 1 | -69 | -88 | 6ACM-d |
| *6-monoacetyl-morphine-d3* | *-* |  | *99.6* | *6.3* | *-* |  | *108.1* | *13.0* | *-* |  | *-* |  | *-* |  | *-72* | *-89* |  |
| 4-androstenedione | 0.9922 | 0.25-100 | 91.3 | 2.6 | 94.2 | 7.9 | 80.9 | 9.6 | 95.0 | 3.3 | 115.1 | 14.1 | 2.5 | 2.5 | -69 | -85 | TEST-d |
| (±)-11-nor-9-Carboxy-Δ9-THC | 0.9918 | 0.2-250 | 18.8 | 46.3 | 73.7 | 28.8 | 28.8 | 25.6 | 100.8 | 6.2 | n.c |  | 80 | 80 | -87 | -80 | THC-COOH-d |
| *(±)-11-nor-9-Carboxy-Δ9-THC -d9* |  |  | 23.6 | 24.5 |  |  | 27.7 | 24.0 |  |  |  |  |  |  | -86 | -82 |  |
| Acetaminophen | 0.9957 | 0.1-500 | 95.1 | 1.68 | 101.3 | 1.5 | 44.8 | 19.4 | 87.0 | 5.1 | n.c. |  | 50 | 50 | -86 | -90 | ACET-d |
| *Acetaminophen-d4* | *-* |  | 92.6 | 3.05 | *-* |  | 43.1 | 11.1 | *-* |  | *-* |  | *-* |  | -84 | -90 |  |
| Acetamiprid | 0.9942 | 0.1-1000 | 91.3 | 1.8 | 120.9 | 3.3 | 100.5 | 1.2 | 77.1 | 1.7 | n.c. |  | 2.5 | 2.5 | -69 | -86 | DEA-d |
| Amphetamine | 0.9962 | 0.1-100 | 94.4 | 1.1 | 107.8 | 2.1 | 19.7 | 7.4 | 106.9 | 1.2 | 107.9 | 1.5 | 1 | 5 | -72 | -66 | AM-d |
| *Amphetamine-d11* |  |  | *88.8* | *2.9* |  |  | *15.3* | *6.2* |  |  |  |  |  |  | *-74* | *-59* |  |
| Atrazine | 0.9991 | 0.05-1000 | 85.0 | 1.3 | 103.9 | 1.9 | 82.0 | 10.5 | 99.0 | 1.1 | 105.9 | 3.8 | 0.5 | 1 | -50 | -66 | ATZ-d |
| *Atrazine-d5* |  |  | *83.9* | *2.7* |  |  | *81.4* | *10.7* |  |  |  |  |  |  | *-51* | *-71* |  |
| Benzophenone-4 | 0.9978 | 0.05-500 | 94.0 | 4.8 | 127.1 | 8.3 | 100.9 | 3.1 | 87.0 | 7.7 | n.c |  | 10 | 10 | -31 | -83 | NPX-d |
| Benzoylecgonine | 0.9948 | 0.05-500 | 86.5 | 1.4 | 92.3 | 3.5 | 121.0 | 9.6 | 57.5 | 5.1 | n.c |  | 5 | 2 | -22 | -87 | BE-d |
| *Benzoylecgonine-d3* | *-* |  | 88.9 | 2.1 | *-* |  | 106.7 | 2.8 | *-* |  | *-* |  | *-* |  |  | -73 |  |
| Bezafibrate | 0.9987 | 0.05-500 | 84.7 | 5.4 | 101.2 | 1.7 | 90.7 | 3.8 | 92.6 | 1.05 | 95.2 | 3.5 | 10 | 5 | 19 | -78 | BZF-d |
| *Bezafibrate-d6* | *-* |  | *82.6* | *7.0* | *-* |  | *91.3* | *4.82* | *-* |  | *-* |  | *-* |  | *19* | *-77* |  |

**Table S3** – (continued)

|  | **Linearity** | | **Accuracy and precision*** | | | | | | | | | | **Sensitivity** | | **Matrix effects** | | **IS used** |
| --- | --- | --- | --- | --- | --- | --- | --- | --- | --- | --- | --- | --- | --- | --- | --- | --- | --- |
|  | R2 | Range  (ng/mL) | 100 mL extraction – 400 ng/L anaytes and 100 ng/L IS | | | | 500 mL extraction  – 700 ng/L analytes + 80 ng/L IS | | | | 500 mL extraction –  7 ng/L analytes + 80 ng/L IS | | 100 mL | 500 mL | 100 mL | 500 mL |  |
|  |  |  | Recovery (%) | RSD  (%) | Trueness  (%) | RSD  (%) | Recovery (%) | RSD  (%) | Trueness  (%) | RSD  (%) | Trueness  (%) | RSD  (%) | LOQ  (ng/L) | LOQ  (ng/L) | % | % |  |
| Caffeine | 0.9937 | 0.01-900 | 93.8 | 0.4 | 101.7 | 5.6 | 91.6 | 12.9 | 109.0 | 3.0 | n.c |  | 5 | 1 | -80 | -91 | CAF-d |
| *Caffeine-d9* | *-* |  | *96.1* | *5.1* | *-* |  | *92.7* | *7.7* | *-* |  | *-* |  | *-* |  | *-83* | *-92* |  |
| Carbamazepine | 0.9961 | 0.01-50 | 90.7 | 1.7 | 91.4 | 3.4 | 96.7 | 7.1 | 90.1 | 4.1 | 98.0 | 12.3 | 1 | 1 | -58 | -66 | ATZ-d |
| Carbendazim | 0.9972 | 0.01-500 | 93.5 | 1.1 | 97.8 | 3.4 | 97.3 | 3.8 | 102.1 | 1.2 | 100.3 | 3.0 | 0.5 | 1 | -71 | -85 | CARB-d |
| *Carbendzim-d3* | *-* |  | *91.6* | *2.9* | *-* |  | *97.2* | *3.2* |  |  | *-* |  | *-* | *-* | *-70* | *-85* |  |
| Clothianidin | 0.9959 | 0.1-500 | 95.5 | 2.2 | 107.4 | 7.8 | 97.8 | 1.9 | 92.5 | 7.9 | 116.1 | 10.3 | 2.5 | 2 | -88 | -93 | CLOT-d |
| *Clothianidin-d3* | *-* |  | *92.8* | *5.8* | *-* |  | *103.0* | *7.6* | *-* |  | *-* |  | *-* | *-* | *-88* | *-94* |  |
| Cocaine | 0.9977 | 0.01-500 | 101.0 | 1.4 | 102.0 | 3.4 | 95.0 | 3.9 | 93.9 | 0.5 | 82.8 | 6.1 | 2 | 2 | -53 | -67 | COC-d |
| *Cocaine-d3* | *-* |  | *98.0* | *3.5* | *-* |  | *95.9* | *4.1* | *-* |  | *-* |  | *-* | *-* | *-53* | *-67* |  |
| Cotinine | 0.9973 | 0.1-500 | 197.2 | 25 | 117.3 | 1.5 | 5.61 | 23 | 115.3 | 1.9 | n.c |  | 25 | 25 | -69 | -34 | COT-d |
| *Cotinine-d3* | *-* |  | *190.5* | *23.8* | *-* |  |  |  | *-* |  | *-* |  | *-* | *-* | *-76* | *-29* |  |
| Desethyl-atrazine | 0.9991 | 0.05-900 | 147.3 | 7.4 | 89.2 | 10.1 | 101.8 | 2.0 | 105.3 | 1.1 | 115.2 | 2.8 | 2.5 | 1 | -87 | -81 | DEA-d6 |
| *Desethyl-atrazine-d6* | *-* |  | 91.1 | 3.6 | *-* |  | 100.5 | 1.3 | *-* |  | *-* |  | *-* | *-* | -77 | -84 |  |
| Desisopropyl-atrazine | 0.9952 | 0.1-900 | 89.9 | 1.4 | 145 | 5.7 | 93.4 | 13.6 | 127.1 | 3.1 | n.c |  | 5 | 8 | -87 | -89 | DIA-d6 |
| *Desisopropyl-atrazine-d5* | *-* |  | 92.3 | 6.4 | *-* |  | 104.4 | 10.5 | *-* |  | *-* |  | *-* | *-* | -76 | -94 |  |
| Diazinon | 0.9989 | 0.1-250 | 47.5 | 6.2 | 103.0 | 1.8 | 27.3 | 11.2 | 125.1 | 3.0 | n.c |  | 0.3 | 0.3 | -40 | -61 | DIAZ-d10 |
| *Diazinon-d10* | *-* |  | 46.7 | 7.1 | *-* |  | 30.0 | 8.9 | *-* |  | *-* |  | *-* | *-* | -39 | -76 |  |
| Dichlorvos | 0.9978 | 25-900 | 54.2 | 32.9 | 96.6 | 38.1 | 70.3 | 7.7 | 130.9 | 11.3 | BLOD |  | 300 | 300 | -80 | -83 | CLOT-d |

**Table S3** – (continued)

|  | **Linearity** | | **Accuracy and precision*** | | | | | | | | | | **Sensitivity** | | **Matrix effects** | | **IS used** |
| --- | --- | --- | --- | --- | --- | --- | --- | --- | --- | --- | --- | --- | --- | --- | --- | --- | --- |
|  | R2 | Range  (ng/mL) | 100 mL extraction – 400 ng/L anaytes and 100 ng/L IS | | | | 500 mL extraction  – 700 ng/L analytes + 80 ng/L IS | | | | 500 mL extraction –  7 ng/L analytes + 80 ng/L IS | | 100 mL | 500 mL | 100 mL | 500 mL |  |
|  |  |  | Recovery (%) | RSD  (%) | Trueness  (%) | RSD  (%) | Recovery (%) | RSD  (%) | Trueness  (%) | RSD  (%) | Trueness  (%) | RSD  (%) | LOQ  (ng/L) | LOQ  (ng/L) | % | % |  |
| Diclofenac | 0.9977 | 0.25-500 | 48.9 | 13.8 | 119.5 | 1.8 | 11.1 | 30.1 | 126.8 | 1.4 | n.c |  | 7 | 20 | -20 | -87 | DICLO-d |
| *Diclofenac-d6* | *-* |  | 48.2 | 12.4 | *-* |  | 8.5 | 38.4 | *-* |  | *-* |  | *-* | *-* | -22 | -88 |  |
| Dimethoate | 0.9951 | 0.1-900 | 90.4 | 1.8 | 140.3 | 5.3 | 102.0 | 7.7 | 139.4 | 2.2 | 164.5 | 7.5 | 5 | 2 | -82 | -87 | CLOT-d |
| Diuron | 0.9938 | 0.1-900 | 82.2 | 5.3 | 101.6 | 2.4 | 86.9 | 12.8 | 89.3 | 2.1 | 94.0 | 4.9 | 2 | 1 | -74 | -83 | DIU-d |
| *Diuron-d6* | *-* |  | *81.6* | *6.8* | *-* |  | *87.5* | *11.3* | *-* |  | *-* | *-* | *-* | *-* | *-75* | *-85* |  |
| Fluometuron | 0.9914 | 0.25-900 | 86.9 | 0.7 | 134.6 | 6.9 | 102.1 | 5.9 | 110.2 | 6.5 | 105.0 | 12.1 | 20 | 20 | -68 | -82 | DIU-d |
| Fluoxetine | 0.9959 | 0.25-100 | 49.02 | 17.9 | 98.4 | 7.4 | 40.7 | 17.5 | 94.5 | 1.3 | 36.9 | 5.0 | 20 | 20 | -60 | -80 | FLX-d |
| *Fluoxetine-d6* | *-* |  | *49.2* | *22.5* | *-* |  | *43.364* | *17.1* | *-* |  | *-* |  | *-* | *-* | *-63* | *-80* |  |
| Furosemide | 0.9974 | 0.05-500 | 72.9 | 3.9 | 70.8 | 13.7 | 80.1 | 4.7 | 42.0 | 1.4 | n.c |  | 50 | 10 | -57 | -89 | NPX-d |
| Gabapentin | 0.9912 | 0.05-500 | 30.3 | 1.8 | 72.3 | 7.3 | 12.4 | 31.0 | 60.7 | 7.7 | nc |  | 10 | 5 | -60 | -75 | ACET-d |
| Gemfibrozil | 0.9961 | 0.05-500 | 57.2 | 16.5 | 110.2 | 6.0 | 58.5 | 14.9 | 65.4 | 9.2 | n.c |  | 2 | 1 | -15 | -81 | DICLO-d |
| Hydrochlorothiazide | 0.9962 | 0.01-50 | 90.6 | 1.5 | 72.9 | 13.2 | 50.4 | 2.8 | 27.6 | 5.7 | n.c |  | 1 | 10 | -68 | -79 | BZF-d |
| Imidacloprid | 0.9952 | 0.01-500 | 93.6 | 4.2 | 80.8 | 7.2 | 100.3 | 5.6 | 102.9 | 8.9 | 132.2 | 23 | 5 | 10 | -82 | -91 | CLOT-d |
| Ketamine | 0.9973 | 0.01-900 | 92.9 | 2.4 | 99.5 | 1.3 | 94.3 | 2.9 | 97.7 | 2.8 | 97.9 | 3.2 | 0.2 | 0.5 | -66 | -77 | KETA-d |
| *Ketamine-d4* | *-* |  | *90.1* | *1.8* | *-* |  | *94.5* | *3.1* | *-* |  | *-* |  | *-* | *-* | *-67* | *-77* |  |
| Ketoprofen | 0.9995 | 0.25-100 | 81.3 | 9.8 | 106.0 | 3.7 | 79.2 | 10.7 | 68.2 | 2.3 | 83.6 | 12.9 | 10 | 6 | -72 | -89 | DIU-d |
| Malathion | 0.9974 | 0.25-250 | 76.1 | 6.1 | 85.8 | 5.1 | 75.4 | 9.7 | 114.7 | 2.7 | 130.2 | 9.1 | 5 | 20 | -51 | -77 | DIU-d |

**Table S3** – (continued)

|  | **Linearity** | | **Accuracy and precision*** | | | | | | | | | | **Sensitivity** | | **Matrix effects** | | **IS used** |
| --- | --- | --- | --- | --- | --- | --- | --- | --- | --- | --- | --- | --- | --- | --- | --- | --- | --- |
|  | R2 | Range  (ng/mL) | 100 mL extraction – 400 ng/L anaytes and 100 ng/L IS | | | | 500 mL extraction  – 700 ng/L analytes + 80 ng/L IS | | | | 500 mL extraction –  7 ng/L analytes + 80 ng/L IS | | 100 mL | 500 mL | 100 mL | 500 mL |  |
|  |  |  | Recovery (%) | RSD  (%) | Trueness  (%) | RSD  (%) | Recovery (%) | RSD  (%) | Trueness  (%) | RSD  (%) | Trueness  (%) | RSD  (%) | LOQ  (ng/L) | LOQ  (ng/L) | % | % |  |
| MDMA | 0.9974 | 0.01-500 | 92.3 | 1.5 | 97.9 | 1.4 | 105.6 | 15.0 | 108.4 | 1.4 | 100.4 | 2.0 | 0.5 | 0.25 | -68 | -92 | MDMA-d |
| *MDMA-d5* | *-* |  | *89.7* | *1.1* | *-* |  | *102.2* | *14.5* | *-* |  | *-* |  | *-* | *-* | *-67* | *-93* |  |
| Methamphetamine | 0.9985 | 0.01-100 | 91.9 | 1.3 | 98.8 | 2.3 | 45.0 | 12.5 | 82.5 | 1.7 | 73.3 | 6.9 | 0.2 | 0.1 | -67 | -84 | MA-d |
| *Methamphetamine-d14* | *-* |  | *92.1* | *2.1* | *-* |  | *37.3* | *10.9* | *-* |  | *-* |  | *-* | *-* | *-68* | *-80* |  |
| Morphine | 0.9904 | 0.1-1000 | 76.8 | 2.2 | 101.9 | 2.1 | 35.2 | 4.1 | 124.5 | 2.1 | 106.1 | 4.4 | 2 | 10 | -28 | 26 | MOR-d |
| *Morphine-d3* | *-* |  | *73.7* | *2.4* | *-* |  | *19.4* | *4.5* | *-* |  | *-* |  | *--* | *-* | *-27* | *31* |  |
| Naproxen | 0.9988 | 0.01-900 | 73.4 | 8.2 | 101.9 | 3.7 | 76.2 | 6.7 | 98.4 | 2.7 | 160.7 | 7.9 | 3 | 1 | -33 | -76 | NPX-d |
| *Naproxen-13C-d3* | *-* |  | *72.0* | *11.3* | *-* |  | *78.6* | *5.5* | *-* |  | *-* |  | *-* | *-* | *-40* | *-76* |  |
| Parathion methyl | 0.9945 | 2.5-500 | 60.8 | 16.7 | 93.3 | 10.9 | 39.1 | 17.4 | 58.6 | 7.9 | n.c |  | 50 | 50 | -67 | -81 | DIU-d |
| Simazine | 0.9979 | 0.05-900 | 87.0 | 0.6 | 101.3 | 2.8 | 93.9 | 5.6 | 98.4 | 6.0 | 99.3 | 5.3 | 0.4 | 0.1 | -54 | -70 | ATZ-d |
| Sulfamethoxazole | 0.9966 | 0.05-500 | 79.9 | 2.7 | 74.2 | 4.3 | 52.9 | 8.1 | 90.0 | 8.2 | 113.6 | 16.2 | 5 | 40 | -81 | -93 | SMX-d |
| *Sulfamethoxazole-d4* | *-* |  | *93.8* | *2.1* | *-* |  |  |  | *-* |  | *-* |  | *-* | *-* | *-79* | *-93* |  |
| Tebuconazole | 0.9961 | 0.1-250 | 69.6 | 8.1 | 106.5 | 0.5 | 53.5 | 15.1 | 96.3 | 0.7 | 81.5 | 13.3 | 1 | 4 | -21 | -52 | TEB-d |
| *Tebuconazole-d6* | *-* |  | *69.4* | *9.9* | *-* |  | *51.7* | *14.8* | *-* |  | *-* |  | *-* | *-* | *-21* | *-56* |  |
| Terbuthylazine-desethyl | 0.9911 | 0.05-900 | 88.5 | 1.0 | 111.9 | 3.4 | 59.1 | 8.2 | 97.7 | 1.1 | 118.2 | 0.7 | 0.2 | 0.1 | -52 | -72 | TERDES-d |
| *Terbuthylazine-desethyl-d9* | *-* |  | *89.5* | *2.9* | *-* |  | *88.8* | *9.5* | *-* |  | *-* |  | *-* | *-* | *-53* | *-77* |  |
| Terbuthylazine | 0.9963 | 0.05-900 | 76.4 | 2.2 | 93.2 | 1.6 | 48.2 | 10.4 | 69.8 | 3.1 | 78.3 | 22.7 | 0.2 | 0.1 | -50 | -61 | ATZ-d |

**Table S3** – (continued)

|  | **Linearity** | | **Accuracy and precision*** | | | | | | | | | | **Sensitivity** | | **Matrix effects** | | **IS used** |
| --- | --- | --- | --- | --- | --- | --- | --- | --- | --- | --- | --- | --- | --- | --- | --- | --- | --- |
|  | R2 | Range  (ng/mL) | 100 mL extraction – 400 ng/L anaytes and 100 ng/L IS | | | | 500 mL extraction  – 700 ng/L analytes + 80 ng/L IS | | | | 500 mL extraction –  7 ng/L analytes + 80 ng/L IS | | 100 mL | 500 mL | 100 mL | 500 mL |  |
|  |  |  | Recovery (%) | RSD  (%) | Trueness  (%) | RSD  (%) | Recovery (%) | RSD  (%) | Trueness  (%) | RSD  (%) | Trueness  (%) | RSD  (%) | LOQ  (ng/L) | LOQ  (ng/L) | % | % |  |
| Testosterone | 0.9947 | 0.1-500 | 85.1 | 3.1 | 95.4 | 7.1 | 82.8 | 10.5 | 107.4 | 4.2 | 79.3 | 10.9 | 5 | 10 | -66 | -75 | TEST-d |
| *Testosterone-d3* |  |  | *82.7* | *9.9* |  |  | *86.0* | *7.0* |  |  |  |  |  |  | *-65* | *-78* |  |
| Theophylline | 0.9952 | 2.5-500 | 92.4 | 0.9 | 123.6 | 6.1 | 78.2 | 6.8 | 75.6 | 3.8 | n.c |  |  |  | -82 | -93 | CAF-d |
| Thiacloprid | 0.9971 | 0.05-250 | 94.6 | 1.4 | 99.7 | 2.4 | 90.4 | 6.3 | 122.7 | 7.2 | 143.0 | 12.3 | 2.50 | 2 | -76 | -89 | CLOT-d |
| Thiamethoxam | 0.9949 | 0.05-1000 | 91.4 | 1.8 | 79.5 | 4.0 | 96.4 | 3.8 | 67.1 | 8.1 | 78.4 | 23.3 | 1 | 1 | -91 | -94 | CLOT-d |
| Triclosan | 0.9989 | 0.1-250 | 16.4 | 34.8 | 144 | 46.9 | 27.3 | 26.6 | 125.8 | 3.6 | 141.7 | 26.3 | 40 | 6 | -71 | -72 | THC-COOH-d |
| Trimetoprim | 0.9966 | 0.01-500 | 91.4 | 1.3 | 98.1 | 1.1 | 93.5 | 2.0 | 96.6 | 2.2 | 97.9 | 3.4 | 1 | 1 | -43 | -66 | TRIM-d |
| *Trimetoprim-d3* | *-* |  | *91.4* | *1.4* | *-* |  | *95.3* | *3.6* | *-* |  | *-* |  | *-* | *-* | *-46* | *-67* |  |

- Linearity is expressed with the linear dynamic range and the R2 value of the linear regression model (excluding the origin, and with a weighting factor of 1/x). Internal standard calibration was used in all cases. For those analytes for which the deuterated analogue was not available, the IS used is indicated in the table *(6ACM-d: 6-monoacetyl-morphine-d3, ACE: acetamiprid; ACET: acetaminophen-d4, AM: amphetamine-d11; ATZ: atrazine-d5; BE: benzoylecgonine-d3; BZF-d: Bezafibrate-d6; BP4: BP4; CAF-d: caffeine-d9; CARB-d: carbendzim-d3, CBMZ: Carbamazepine; CLOT-d: clothianidin-d3; COC-d: cocaine-d3; COT-d: cotinine-d3; DEA-d: desethyl-atrazine-d6; DIA-d: desisopropyl-atrazine-d5; DIAZ-d: diazinon-d10; DICLO-d: diclofenac-d6; DIU-d: diuron-d6; DMT: Dimethoate; FLX-d: fluoxetine-d6; GBP: gabapentin; GFB: Gemfibrozil; IMI: imidacloprid; KETA-d: ketamine-d4; KETO: Ketoprofen; MDMA-d: MDMA-d5; MA-d: methamphetamine-d14; MOR-d: morphine-d3; NPX-d: naproxen-13C-d3; SMZ: simazine; SMX-d: sulfamethoxazole-d4; TBZ: Terbuthylazine; TEB-d: tebuconazole-d6; TERBDES-d: terbuthylazine-desethyl-d9; THC-COOH-d: (±)-11-nor-9-carboxy-Δ9-tetrahydrocannabinol -d9, TEST-d: testosterone-d3; TRIM-d: trimethoprim-d3).*
- Analyte recovery was calculated by comparing the peak areas obtained in the LC-MS/MS analysis of samples fortified at equivalent concentrations with the target analytes and deuterated compounds at the beginning and at the end of the sample processing protocol (see tested concentrations in the table) (n=3) (background signal if existing was subtracted from the signals observed).
- RSD: relative standard deviation of the analyte recoveries or trueness values (n=3)
- Trueness was calculated by comparing the calculated concentration and the expected concentration and expressed in %.
- Sensitivity, the limit of quantification of the method was estimated from the samples analyzed and if the compound was not present in any sample, from the samples used in the recovery study, as the water concentration that would provide a signal 10 times higher than the signal-to-noise ratio observed in the samples.
- n.c. – not calculated due to the elevated background concentrations in the sample used in the recovery study.
- Values in red colour indicate that the level of the recovery study is out of the analyte linearity range.

**Table S4** Comparison of LOQs and freshwater PNECs used for the environmental risk assessment. Values < 1 indicate that the analytical LOQ was below the corresponding PNEC.

| **Compound** | **LOQ**  **(ng L^-1^)** | **PNEC**  **(ng L⁻¹)^a^** | **AF** | **Target taxon** | **Derivation method** | **Source** | **LOQ / PNEC** |
| --- | --- | --- | --- | --- | --- | --- | --- |
| Acetaminophen | 50 | 46000 | 10 | n.r. | Deterministic | FASS (2022) | < 1 |
| Acetamiprid | 2.5–2.5 | 37 | 0 | n.r. | n.r. | FASS (2022) | < 1 |
| Amphetamine | 1–5 | 24800 | 1000 | Fish | Deterministic | Aalizadeh et al. (2017) | < 1 |
| Atrazine | 0.5–1 | 100 | 0 | n.r. | n.r. | EU Directive (2024) | < 1 |
| Benzoylecgonine | 2–5 | 2330 | n.r. | n.r. | n.r. | López-García et al. (2021)* | < 1 |
| BP-4 (Sulisobenzone) | 10 | 3520 | 1000 | Fish | Deterministic | Aalizadeh et al. (2017) | < 1 |
| Caffeine | 1–5 | 1200 | 100 | *Daphnia magna* | Deterministic | UBA (2017) | < 1 |
| Carbamazepine | 1 | 2500 | 0 | n.r. | n.r. | European Commission (2024) | < 1 |
| Carbendazim | 0.5–1 | 440 | 10 | *Daphnia magna* | Deterministic | Oekotoxzentrum (2016) | < 1 |
| Cocaine | 2 | 2460 | 1000 | Fish | Deterministic | Aalizadeh et al. (2017) | < 1 |
| Cotinine | 25 | 10000 | 100 | *Lemna gibba* | Deterministic | US EPA (2018) | < 1 |
| Desethyl-atrazine | 1–2.5 | 260 | 1000 | Algae and plants | Deterministic | Aalizadeh et al. (2017) | < 1 |
| Desisopropyl-atrazine | 5–8 | 390 | 1000 | Algae and plants | Deterministic | Aalizadeh et al. (2017) | < 1 |
| Diazinon | 0.3 | 10 | 50 | *Daphnia magna* | Deterministic | UBA (2016) | < 1 |
| Diclofenac | 7–20 | 40 | 0 | n.r. | n.r. | European Commission (2024) | < 1 |
| Dimethoate | 2–5 | 70 | 100 | *Baetis rhodani* | Deterministic | Oekotoxzentrum (2012) | < 1 |
| Diuron | 1–2 | 49 | 0 | n.r. | n.r. | European Commission (2024) | < 1 |
| Gabapentin | 5–10 | 1000000 | 100 | Plants and invertebrates | Deterministic | Wünnemann et al. (2020) | < 1 |

**Table S4** – (continued)

| **Compound** | **LOQ**  **(ng L^-1^)** | **PNEC**  **(ng L⁻¹)^a^** | **AF** | **Target taxon** | **Derivation method** | **Source** | **LOQ / PNEC** |
| --- | --- | --- | --- | --- | --- | --- | --- |
| Gemfibrozil | 1–2 | 500 | 10 | *Danio rerio* | Deterministic | US EPA (2018) | < 1 |
| Imidacloprid | 5–10 | 6.8 | 0 | n.r. | n.r. | European Commission (2024) | ≈ 1 |
| Ketamine | 0.2–0.5 | 5710 | 1000 | Crustaceans | Deterministic | Aalizadeh et al. (2017) | < 1 |
| Ketoprofen | 6–10 | 2100 | 1000 | Fish | Deterministic | Aalizadeh et al. (2017) | < 1 |
| MDMA (3,4-Methylenedioxymethamphetamine) | 0.25–0.5 | 30010 | 1000 | Algae and plants | Deterministic | Aalizadeh et al. (2017) | < 1 |
| Methamphetamine | 0.1–0.2 | 9740 | 1000 | Crustaceans | Deterministic | Aalizadeh et al. (2017) | < 1 |
| Morphine | 2–10 | 5380 | 1000 | Algae and plants | Deterministic | Aalizadeh et al. (2017) | < 1 |
| Naproxen | 1–3 | 1700 | 50 | *Ceriodaphnia dubia* | Deterministic | Oekotoxzentrum (2015) | < 1 |
| Simazine | 0.1–0.4 | 1000 | 0 | n.r. | n.r. | European Commission (2024) | < 1 |
| Sulfamethoxazole | 5–40 | 100 | 0 | n.r. | n.r. | European Commission (2022) | < 1 |
| Tebuconazole | 1–4 | 240 | 0 | n.r. | n.r. | European Commission (2022) | < 1 |
| Terbuthylazine | 0.1–0.2 | 220 | 10 | *Scenedesmus vacuolatus* | Deterministic | Oekotoxzentrum (2016) | < 1 |
| Trimethoprim | 1 | 100 | 0 | n.r. | n.r. | European Commission (2022) | < 1 |
| Terbuthylazine-desethyl | 0.1–0.2 | 250 | 0 | n.r. | n.r. | RIVM (2018) | < 1 |

LOQ: Limit of Quantification.

^a^PNEC: Predicted No-Effect Concentration for freshwater organisms extracted from the NORMAN Ecotoxicology Database unless otherwise indicated.

AF, target taxon and derivation method are reported as provided by the NORMAN Ecotoxicology Database (n.r. = not reported). For regulatory environmental quality standards (EQS), AF and derivation method are not provided.

*For benzoylecgonine, the freshwater PNEC (2.33 µg L⁻¹) corresponds to López-García et al. (2021), who extracted this value from a previous version of the NORMAN Ecotoxicology Database. The value is no longer available in the current version.

**Fig. S1** Percentage of occurrence of ECs in the sampling stations during wet-season pond-system flow (A), dry-season pond-system flow (B) and temporary pond-system bypas (C).

**Table S5** RQ_sample_ and standard deviations in the sampling stations during wet-season pond-system flow, dry-season pond-system flow and temporary pond-system bypass. Different lowercase letters indicate significant differences between sampling stations (*Q-*value < 0.05)

|  | **Wet-season pond-system flow** | | | | |
| --- | --- | --- | --- | --- | --- |
|  | **Station A** | **Station A** | **Station A** | **Station D** | **Station E** |
| ***Pesticides*** | 23.09 ± 11.65^a^ | 6.91 ± 2.85^bc^ | 8.50 ± 7.29^bd^ | 4.87 ± 5.84^ce^ | 2.98 ± 1.74^de^ |
| ***Stimulants and illicit drugs*** | 6.07 ± 5.11^a^ | 0.62 ± 0.42^a^ | 0.62 ± 0.21^a^ | 0.32 ± 0.15^a^ | 0.60 ± 0.55^a^ |
| **PhPCPs** | 2.53 ± 0.56^a^ | 0.74 ± 0.32^b^ | 0.84 ± 0.21^b^ | 0.38± 0.13^c^ | 0.46 ± 0.25^c^ |
|  | **Dry-season pond-system flow** | | | | |
|  | **Station A** | | **Station B** | **Station C** | |
| ***Pesticides*** | 18.77 ± 19.84^a^ | | 8.48 ± 8.08^b^ | 6.29 ± 6.72^c^ | |
| ***Stimulants and illicit drugs*** | 4.77 ± 3.55^a^ | | 0.49 ± 0.44^b^ | 0.28 ± 0.18^b^ | |
| **PhPCPs** | 2.33 ± 0.95^a^ | | 0.74 ± 0.34^b^ | 0.72 ± 0.31^b^ | |
|  | **Temporary pond-system bypass** | | | | |
|  | **Station A’** | | **Station B’** | **Station E’** | |
| ***Pesticides*** | 4.89 ± 1.32^a^ | | 4.23 ± 1.00^a^ | 4.55 ± 0.63^a^ | |
| ***Stimulants and illicit drugs*** | 1.52 ± 1.78^a^ | | 0.62 ± 0.71^a^ | 0.56 ± 0.56^a^ | |
| **PhPCPs** | 3.21 ± 1.37^ab^ | | 2.97 ± 1.13^a^ | 2.72 ± 1.11^b^ | |

**Text S1** Calculation of removal efficiencies between stations A and B

ECs removal (%) between stations A (WWTP effluent channel) and B (Laguneto outlet channel) was computed with the mass-balance expression of Matamoros et al. (2015), adapted to field conditions:

$$\text{Removal (\%)}=\frac{1}{n}\sum_{i=1}^{n} \frac{C_{A}^{'}-\left( C_{B,i}-C_{B,i}\frac{EVR_{i}-P_{i}}{HLR_{i}} \right)}{C_{A}^{'}}\times100$$

C’_A_ is the sampling-average influent concentration (station A), C_B,i_ is the effluent concentration on sampling day i, and n=6 and n=15 is the number of paired samples per hydraulic scenario, wet- and dry-season pond-system flow periods respectively. The hydraulic loading rate (HLR_i_, L m⁻² d⁻¹) was derived daily from the WWTP flow divided by the free-water surface of Laguneto, while open-water evaporation (EVR_i_, L m⁻² d⁻¹) was estimated with Turc’s equation and converted with a calibrated pan coefficient (0.80). Daily rainfall (P_i_, L m⁻² d⁻¹) measured at the on-site weather station was subtracted from EVR to account for dilution; the resulting dimensionless factor (EVR_i_,-P_i_)/HLR_i_ corrects C_B,i_ for net atmospheric water exchange before removal is expressed as a percentage of C’_A_. Rain and evaporation corrections were based on Laguneto water surface área, while additional minor water inputs or losses were assumed negligible.

**Fig. S2** Removal efficiency (%) of individual ECs between station A (WWTP effluent channel) and station B (Laguneto outlet channel) during the dry- and wet-season pond-system flow periods (left and right panels, respectively).  Boxes show the inter-quartile range, horizontal lines mark the median, whiskers extend to 1.5 × IQR, and points are individual paired samples (n = 8).

**Table S6** Physicochemical properties and half-lives for target ECs analyzed in this study.

| **Compound** | **Solubility (mg L^-1^)** | **logKow^*^** | **DT50 (days)** | **DT50 references** |
| --- | --- | --- | --- | --- |
| Acetaminophen | 1.43 × 10⁴ | 0.46 | 20-30 | 1,2 |
| Acetamiprid | 4200 | 1.51 | 4.7 | EPA |
| Amphetamine | 7300 | 1.65 | 3 | 3 |
| Atrazine | 34 | 2.61 | 30-100 | 4 |
| Benzoylecgonine | 1610 | 1.57 | 36.7 | 5 |
| BP-4 | 250 | 1.23 | 0.2-60.7 | 6 |
| Caffeine | 2.16 × 10⁴ | -0.07 | 100-240 | 7,8 |
| Carbamazepine | 17.7 | 2.45 | 82 | 9 |
| Carbendazim | 8 | 1.52 | 60 | EPA |
| Cocaine | 1670 | 2.30 | 0.6-2 | EPA |
| Cotinine | 1000 | 0.70 | 1-2 | EPA |
| Desethyl-atrazine | 110 | 1.51 | 2.23 | 10 |
| Desisopropyl-atrazine | 90 | 1.15 | NA |  |
| Diazinon | 40 | 3.81 | 4.3 | 11 |
| Diclofenac | 2.4 | 4.51 | 8 | 2 |
| Dimethoate | 3.9 x 10⁴ | 0.78 | 12.6 | 11 |
| Diuron | 42 | 2.68 | 8.8 | 11 |
| Gabapentin | 5.8 x 10⁴ | -1.1 | 2-7 | 12 |
| Gemfibrozil | 10 | 4.44 | 70-128 | 13 |
| Imidacloprid | 610 | 0.57 | 30 | 11 |
| Ketamine | 2 x 10⁴ | 2.40 | 0.5 | 14 |
| Ketoprofen | 51 | 3.12 | 4.75-9.97 | 15 |
| MDMA | 10 x 10^5^ | 2.15 | NA |  |
| Methamphetamine | 19 x 10^5^ | 2.07 | 1.75 | 3 |
| Morphine | 60 x 10^4^ | 0.89 | NA |  |
| Naproxen | 15 | 3.18 | 14 | 11 |
| Simazine | 6.2 | 2.18 | 46 | 11 |
| Sulfamethoxazole | 610 | 0.89 | 6.6-13 | 9 |
| THC-COOH | 28 | 5.73 | NA |  |
| Trimethoprim | 400 | 0.91 | 15 | 9 |
| Tebuconazole | 36 | 3.70 | 90 | 10 |
| Terbuthylazine | 8 | 3.21 | 6 | 11 |
| Terbutylazine-desethyl | 40 | 1.94 | NA |  |

Solubility: solubility in water at at 25 °C and neutral pH;

^*^logKow: octanol-water partition coefficient

DT50: degradation potential in water phase, expressed as half-life in days

EPA: U.S. Environmental Protection Agency. (2025). CompTox Chemicals Dashboard.

Retrieved August 10, 2025, from https://comptox.epa.gov/dashboard/chemical/properties/DTXSID0020074

1. Peuravuori J., 2012. Aquatic photochemistry of paracetamol in the presence of dissolved organic chromophoric material and nitrate. *Environ. Sci. Pollut. Res.* 19, 2259-2270.
2. Bouissou-Schurtz, C., Houeto, P., Guerbet, M., Bachelot, M., Casellas, C., Mauclaire, A.-C., Panetier, P., Delval, C., Masset, D., 2014. Ecological risk assessment of the presence of pharmaceutical residues in a French national water survey. *Regul. Toxicol. Pharmacol.* 69, 296– https://doi.org/10.1016/j.yrtph.2014.04.006
3. Lee, S.S., Paspalof, A.M., Snow, D.D., Richmond, E.K., Rosi-Marshall, E.J., Kelly, J.J., 2016. Occurrence and potential biological effects of amphetamine on stream communities. *Environ. Sci. Technol.* 50, 9727–9735. <https://doi.org/10.1021/acs.est.6b03717>
4. Wu, S., Li, H., Li, X., He, H., Yang, C., 2018. Performances and mechanisms of efficient degradation of atrazine using peroxymonosulfate and ferrate as oxidants. *Chem. Eng. J.* 353, 533–541. <https://doi.org/10.1016/j.cej.2018.06.133>.
5. Salcedo, G., Niroula, V., Pagsuyoin, S.A., 2025. Adverse impacts of drugs of abuse on the survival, growth, and mobility of *C. virginica* oyster larvae. *Mar. Pollut. Bull.* 216, 117985. <https://doi.org/10.1016/j.marpolbul.2025.117985>.
6. Semones, M.C., Sharpless, C.M., MacKay, A.A., Chin, Y.-P., 2017. Photodegradation of UV filters oxybenzone and sulisobenzone in wastewater effluent and by dissolved organic matter. *Appl. Geochem.* 83, 150–157. <https://doi.org/10.1016/j.apgeochem.2017.02.0>
7. Hillebrand O., Nodler K., Licha T., Sauter M., Geyer T., 2012. Caffeine as an indicator for the quantification of untreated wastewater in karst systems. *Water Res.* 46, 395–402. <https://doi.org/10.1016/j.watres.2011.11.003>
8. Buerge I.J., Poiger, T., Müller, M.D., Buser, H.-R., 2006. Combined sewer overflows to surface waters detected by the anthropogenic marker caffeine. *Environ. Sci. Technol.* 40, 4096–102. https://doi.org/10.1021/es052553l
9. Lam M.W., Young C.J., Brain R.A., Johnson D.J., Hanson M.A., Wilson C.J., Richards, S.M., Solomon, K.R., Mabury, S.A., 2004. Aquatic persistence of eight pharmaceuticals in a microcosm study. *Environ. Toxicol. Chem.* 23, 1431–40. <https://doi.org/10.1897/03-421>
10. Barbieri, M.V., Peris, A., Postigo, C., Moya-Garcés, A., Monllor-Alcaraz, L.S., Rambla-Alegre, M., Eljarrat, E., López de Alda, M., 2021. Evaluation of the occurrence and fate of pesticides in a typical Mediterranean delta ecosystem (Ebro River Delta) and risk assessment for aquatic organisms. *Environ. Pollut.* 274, 115813, <https://doi.org/10.1016/j.envpol.2020.115813>
11. PPDB, Pesticide Properties Database. University of Hertfordshire. https://sitem.herts.ac.uk/aeru/ppdb/ Lewis, K.A., Tzilivakis, J., Warner, D. and Green, A., 2016. An international database for pesticide risk assessments and management. *Hum. Ecol. Risk Assess.* 22, 1050–1064.
12. Henning, N., Kunkel, U., Wick, A., Ternes, T., 2018. Biotransformation of gabapentin in surface water matrices under different redox conditions and the occurrence of one major TP in the aquatic environment. *Water Res.* 137, 290–300. [https://doi.org/10.1016/j.watres.2018.01. 027](https://doi.org/10.1016/j.watres.2018.01.%20027).
13. Grenni, P., Patrolecco, L., Ademollo, N., Di Lenola, M., Barra Caracciolo, A., 2018. Assessment of gemfibrozil persistence in river water alone and in co-presence of naproxen, *Microchem. J.* 136, 49–55, <https://doi.org/10.1016/j.microc.2016.11.018>.
14. Lin, A.Y.-C., Lee, W.-N., Wang, X.-H., 2014. Ketamine and the metabolite norketamine: persistence and phototransformation toxicity in hospital wastewater and surface water. *Water Res.* 53, 351–360. https://doi.org/10.1016/j.watres.2014.01.022
15. Araujo L, Troconis, M.E., Espina, M.B., Prieto, A., 2014. Persistence of ibuprofen, ketoprofen, diclofenac and clofibric acid in natural waters. *J. Environ. Hum*. 1, 32–8.

**Fig. S3** Relationship between log_10_ half-life (DT50, days) of ECs and removal (%) in Laguneto, from station A to station B, during wet-season pond-system flow (A) and dry-season pond-system flow (B), and percentage reduction (%) from station A’ to station B’ during temporary pond-system bypass (C). Each point represents the log_10_ of the mean DT50 (average of reported minimum and maximum values) of an individual compound, colored by chemical group (Stimulants and illicit drugs, PhPCPs, Pesticides). Annotated correlation coefficients correspond to Pearson’s r or Spearman’s ρ, depending on whether model assumptions were met, with associated *P*-values.

**Fig. S4** Relationship between compound hydrophobicity (logKow) of ECs and removal (%) in Laguneto, from station A to station B, during wet-season pond-system flow (A) and dry-season pond-system flow (B), and percentage reduction (%) from station A’ to station B’ during temporary pond-system bypass (C). Each point represents the logKov of an individual compound, colored by chemical group (pesticides, stimulants and illicit drugs, PhPCP). Annotated correlation coefficients correspond to Pearson’s *r* or Spearman’s ρ, depending on whether model assumptions were met, with associated *P*-values.

**References**

López-García, E., Mastroianni, N., Ponsà-Borau, N., Barceló, D., Postigo, C., & de Alda, M. L. (2021). Drugs of abuse and their metabolites in river sediments: Analysis, occurrence in four Spanish river basins and environmental risk assessment. *Journal of hazardous materials*, 401, 123312. https://doi.org/10.1016/j.jhazmat.2020.123312

Matamoros, V., Gutiérrez, R., Ferrer, I., García, J., & Bayona, J. M. (2015). Capability of microalgae-based wastewater treatment systems to remove emerging organic contaminants: A pilot-scale study. *Journal of Hazardous Materials*, *288*, 34–42. https://doi.org/10.1016/J.JHAZMAT.2015.02.002
